# Optical pulse labeling studies reveal exogenous seeding slows α-synuclein clearance

**DOI:** 10.1038/s41531-022-00434-4

**Published:** 2022-12-19

**Authors:** Cara L. Croft, Giavanna Paterno, Ava R. Vause, Lyla A. Rowe, Daniel H. Ryu, Marshall S. Goodwin, Corey A. Moran, Pedro E. Cruz, Benoit I. Giasson, Todd E. Golde

**Affiliations:** 1 UK Dementia Research Institute, UCL Institute of Neurology, University College London, London, UK; 2 Department of Neuroscience, College of Medicine, University of Florida, Gainesville, FL USA; 3 Center for Translational Research in Neurodegenerative Disease, College of Medicine, University of Florida, Gainesville, FL USA; 4 McKnight Brain Institute, College of Medicine, University of Florida, Gainesville, FL USA; 5 The Francis Crick Institute, London, UK; 6 Department of Pharmacology and Chemical Biology, Department of Neurology, Emory Center for Neurodegenerative Disease, Emory University, Atlanta, GA USA

**Keywords:** Parkinson's disease, Parkinson's disease, Neurodegeneration

## Abstract

The accumulation of α-synuclein (α-syn) in intracellular formations known as Lewy bodies (LBs) is associated with several neurodegenerative diseases including Parkinson’s disease and Lewy Body Dementia. There is still limited understanding of how α-syn and LB formation is associated with cellular dysfunction and degeneration in these diseases. To examine the clearance and production dynamics of α-syn we transduced organotypic murine brain slice cultures (BSCs) with recombinant adeno-associated viruses (rAAVs) to express Dendra2-tagged human wild-type (WT) and mutant A53T α-syn, with and without the addition of exogenous α-syn fibrillar seeds and tracked them over several weeks in culture using optical pulse labeling. We found that neurons expressing WT or mutant A53T human α-syn show similar rates of α-syn turnover even when insoluble, phosphorylated Ser129 α-syn has accumulated. Taken together, this data reveals α-syn aggregation and overexpression, pSer129 α-syn, nor the A53T mutation affect α-syn dynamics in this system. Prion-type seeding with exogenous α-syn fibrils significantly slows α-syn turnover, in the absence of toxicity but is associated with the accumulation of anti-p62 immunoreactivity and Thiazin Red positivity. Prion-type induction of α-syn aggregation points towards a potential protein clearance deficit in the presence of fibrillar seeds and the ease of this system to explore precise mechanisms underlying these processes. This system facilitates the exploration of α-syn protein dynamics over long-term culture periods. This platform can further be exploited to provide mechanistic insight on what drives this slowing of α-syn turnover and how therapeutics, other genes or different α-syn mutations may affect α-syn protein dynamics.

## Introduction

The accumulation of misfolded α-synuclein (α-syn) in neurons and glia of the central nervous system (CNS) is a feature of the neurodegenerative conditions, collectively known as α-Synucleinopathies. α-Synucleinopathies are multi-faceted diseases with broad physiological and pathological manifestations and include Parkinson’s disease (PD), Lewy Body Dementia (LBD) and Multiple system Atrophy (MSA)^1,2^. Despite the large clinical heterogeneity between these diseases, amyloidogenic fibrillary α-syn aggregates which are heavily phosphorylated at Ser129 (pSer129) are the prominent marker of Lewy bodies (LBs) or Lewy neurites (LNs); found typically in neurons and highlighted as the hallmark pathology in these diseases^3–6^. α-syn is implicated pathologically, genetically and through modeling studies as a driver of disease in these neurodegenerative conditions^7–9^. However, more recent work highlights the highly complex nature and composition of LBs containing an intricate mixture of various aggregated and post-translationally modified forms of α-syn, as well as, hundreds of other proteins, lipids, and disrupted membranes and membranous organelles^10,11^. Therefore, the role of α-syn plus these other factors in the process of LB formation and α-syn aggregation remains less clearly defined. Furthermore, much clinical targeting of the α-Synucleinopathies relies on strategies to reduce α-syn aggregation or production, inhibit LB formation or prevent the ‘prion-like’ spread of α-syn^12,13^, based on several studies that indicate that α-syn contributes to the progression of pathology^14^. Therefore, understanding how aggregation, phosphorylation, insolubility and seeding may affect the overall clearance and production of α-syn is relevant to the development of future therapeutics which seem to highly rely on α-syn aggregation and spread being the major drivers of disease.

We have recently shown that rAAV overexpression of human WT and A53T α-syn in organotypic murine BSCs drives the formation of insoluble, phosphorylated and fibrillar α-syn inclusions^15^, recapitulating features of human LB-like inclusions^1,3^. This provides a rapid and accessible platform to investigate human α-syn and LBs in a cytoarchitecturally and regionally relevant environment. To extend these findings to investigate α-syn protein clearance and production dynamics we developed Dendra2-tagged human WT and mutant A53T α-syn rAAVs and expressed them in BSCs, with and without the addition of exogenous α-syn pre-formed fibrils (PFFs). Using optical pulse-chase labeling (OPL) methodology we identified that both soluble and insoluble, phosphorylated WT and mutant A53T human α-syn, show similar rates of production and turnover to Dendra2 alone. However, upon addition of α-syn PFFs some neurons show a slowing of α-syn turnover, and this is associated with the accumulation of p62 and Thiazin Red positivity, in the absence of any toxicity. Taken together, these findings point towards a potential α-syn clearance deficit in neurons in the presence of PFFs and the utility of this system to explore precise mechanisms underlying protein dynamics and these clearance changes. Furthermore, the data underscores that overexpression and accumulation of insoluble pSer129 α-syn do not necessarily affect the rates of α-syn production and turnover, at least in the period when this LB-like pathology has initially formed. However, the application of exogenous α-syn PFFs slows down the rate of α-syn turnover in neurons in these BSC models and may contribute to disease pathology when such seed-competent species accumulate in the brain. Overall, this system provides a rapid means to investigate modifiers of α-syn protein dynamics and may inform future therapeutic targeting of α-Synucleinopathies.

## Results

### WT and A53T human α-syn turnover at similar rates when soluble in BSC models

**Fig. 1:**
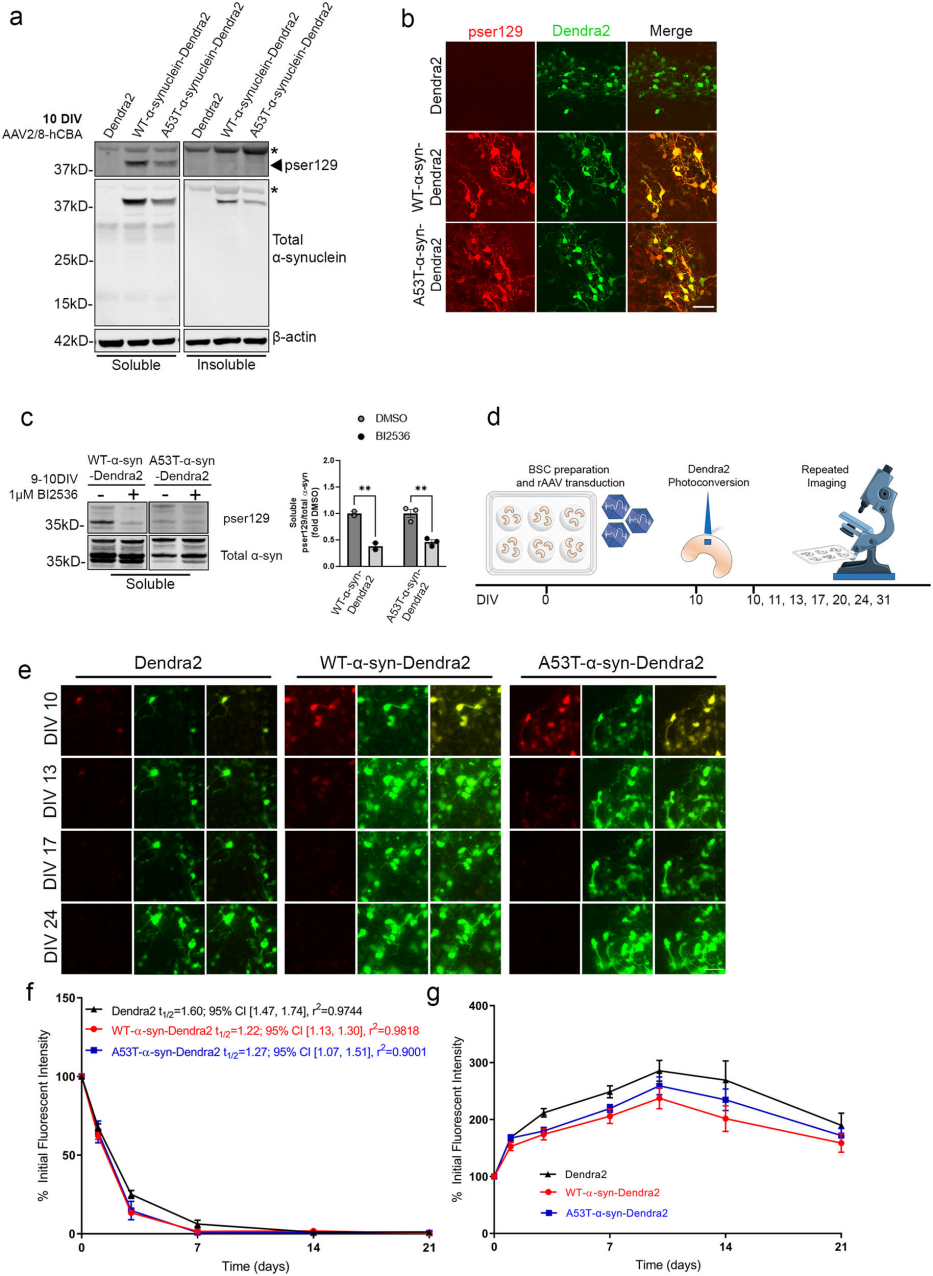
WT and A53T-α-syn-Dendra2 show similar production and turnover rates when soluble in BSCs. BSCs were transduced at 0 DIV with rAAVs to express Dendra2, WT-α-syn-Dendra2 or A53T-α-syn-Dendra2 and maintained in culture until 10 DIV. BSCs were sequentially extracted to prepare soluble and triton-insoluble fractions. **a** Lysates were analyzed on western blots for pSer129 α-syn (81 A) or total α-syn and β-actin as a loading control. The mobility of molecular mass markers are shown on the left. Black arrow indicates pSer129, asterisks indicate non-specific bands. (*n* = 3). **b** BSCs were fixed, immunostained for pSer129 α-syn (EP1536Y) and confocal imaged to confirm the distribution of phosphorylated α-syn. Scale bar = 50 µm. (*n* = 6). **c** BSCs were treated with 1 μM BI2536 or DMSO control for 24 h from 9-10 DIV to reduce levels of soluble pSer129. Representative blots of pSer129 α-syn (EP1536Y) and total α-syn are shown. The mobility of molecular mass markers are shown on the left. Bar chart shows levels of soluble pSer129 α-syn are reduced with BI2536 treatment (*n* = 2-3, data are mean ± SEM, analyzed by two-way ANOVA with post-hoc Bonferroni’s test). **d** Schematic diagram shows the timeline of the long-term optical pulse labeling experiments using photoconversion of Dendra2, WT-α-syn-Dendra2 and A53T-α-syn-Dendra2 at 10 DIV. **e** Representative images of photoconverted (red) and newly synthesized (green) Dendra2 above residual fluorescence in BSCs photoconverted at 10 DIV and imaged at several time points until 31 DIV. Merge of both channels is also shown. Scale bar = 50 µm **f** Line graph shows quantification of population levels of red photoconverted Dendra2 fluorescent intensity in each condition that were quantified over time as a proportion of initial fluorescent intensity (*n* = 12, data are mean ± SEM, analyzed by Two-way ANOVA with post-hoc Sidak’s test). **g** Line graphs show quantification of population levels of green Dendra2 fluorescent intensity in each condition that were quantified over time as a proportion of initial fluorescent intensity (*n* = 12, data are mean ± SEM, analyzed by Two-way ANOVA with post-hoc Sidak’s test).

We have previously shown rAAV-mediated expression of human WT and A53T α-syn rapidly induces LB-like phosphorylated and insoluble α-syn pathology in BSCs by 28 DIV (18). To investigate the protein dynamics underlying human α-syn with and without the A53T PD-related mutation we used the photoswitchable properties of the Dendra2 fluorescent protein (21, 23) in long-term BSCs. We generated rAAVs of Dendra2 alone, and Dendra2 fused to the C-terminus of human WT and A53T α-syn. To characterize the timeline of the development of phosphorylated and insoluble α-syn in BSCs that were transduced with Dendra2, WT-α-syn-Dendra2 and A53T α-syn-Dendra2 we performed biochemical and immunohistochemical assessments initially at 10 DIV. At 10 DIV, overexpression of human α-syn was confirmed in BSCs expressing WT-α-syn-Dendra2 and A53T-α-syn-Dendra2 (Fig. 1a). At this time point soluble pSer129 α-syn was detected but not insoluble pSer129 α-syn. We also identified no increase in 14 kDa α-syn which may occur if the Dendra2 tag was being cleaved from α-syn (Fig. 1a). Distribution of pSer129 soluble α-syn was also assessed by immunohistochemistry with some pSer129 accumulating in WT-α-syn-Dendra2 and A53T-α-syn-Dendra2 transduced BSCs (Fig. 1b). Recent studies have suggested a useful tool to distinguish between insoluble and soluble pSer129 α-syn is to reduce the levels of soluble pSer129 using the PLK1–3 inhibitor, BI2536^16^. We treated 9 DIV BSCs for 24 h until 10DIV with BI2536 and saw a reduction in soluble pSer129 to further characterize the α-syn properties at this stage (Fig. 1c). In 10 DIV BI2536 treated BSCs, insoluble pSer129 α-syn did not accumulate and only limited soluble pSer129 was detected. These studies, and our previous work on tau^17^ provided initial proof of concept that it may be feasible to examine the kinetics of soluble, overexpressed WT and A53T α-syn in optical pulse labeling (OPL) studies in rAAV transduced BSCs.

Dendra2 is a photoswitchable fluorescent protein which enables the tracking of new and old protein populations by OPL in transduced neural cells after photoconversion with short wavelength light. After exposure to short wavelength light, red fluorescence is emitted and then green fluorescence is reduced. New proteins synthesized after the photoconversion emit green fluorescence above residual non-converted green fluorescence enabling the assessment of α-syn dynamics^18,19^.

It is plausible that the contribution of α-syn mutations or overexpression to disease could be through modulation of protein degradation pathways or altering protein stability. Here we examined the longevity and fate of soluble WT and A53T α-syn by conducting long-term OPL experiments to estimate the half-life of α-syn in these conditions. A schematic diagram of the long-term imaging timeline of predominantly soluble α-syn is shown in Fig. 1d. Long-term live imaging for 10 to 31 DIV was used to track the old red fluorescent photoconverted Dendra2-tagged α-syn and the newly synthesized non-photoconverted green, fluorescent Dendra-2 tagged α-syn (Fig. 1e). Upon population level fluorescent density quantification, no differences in amounts of red photoconverted WT and A53T α-syn were identified (Fig. 1f). When estimating half-lives from this data; Dendra2 alone shows a half-life of 1.60 days; 95% CI [1.47, 1.74], r^2^ = 0.9744, WT-α-syn a half-life of 1.22 days; 95% CI [1.13, 1.30], r^2^ = 0.9818 and A53T-α-syn has an estimated half-life of 1.27 days; 95% CI [1.07, 1.51], r^2^ = 0.9001. These results indicate that soluble WT and A53T α-syn turn over at a similar rate as Dendra2 alone. These findings also corroborate previous reports in cell lines and mouse primary neurons where soluble overexpressed human α-syn has a half-life of ~1-2 days^20–22^. Alternatively, mutations or overexpression of α-syn may contribute to disease through modulating α-syn production rates. We, therefore, tracked the production of WT and A53T α-syn by quantifying total green fluorescence emitted above residual fluorescence which increases over time due to new protein synthesis after photoconversion. There were no changes in the production of α-syn as detected using long-term live imaging until 31 DIV (Fig. 1g). Dendra2 alone, WT and A53T α-syn all increased at a similar rate until stabilization.

### Insoluble and phosphorylated WT and A53T α-syn show similar rates of turnover in BSCs

**Fig. 2:**
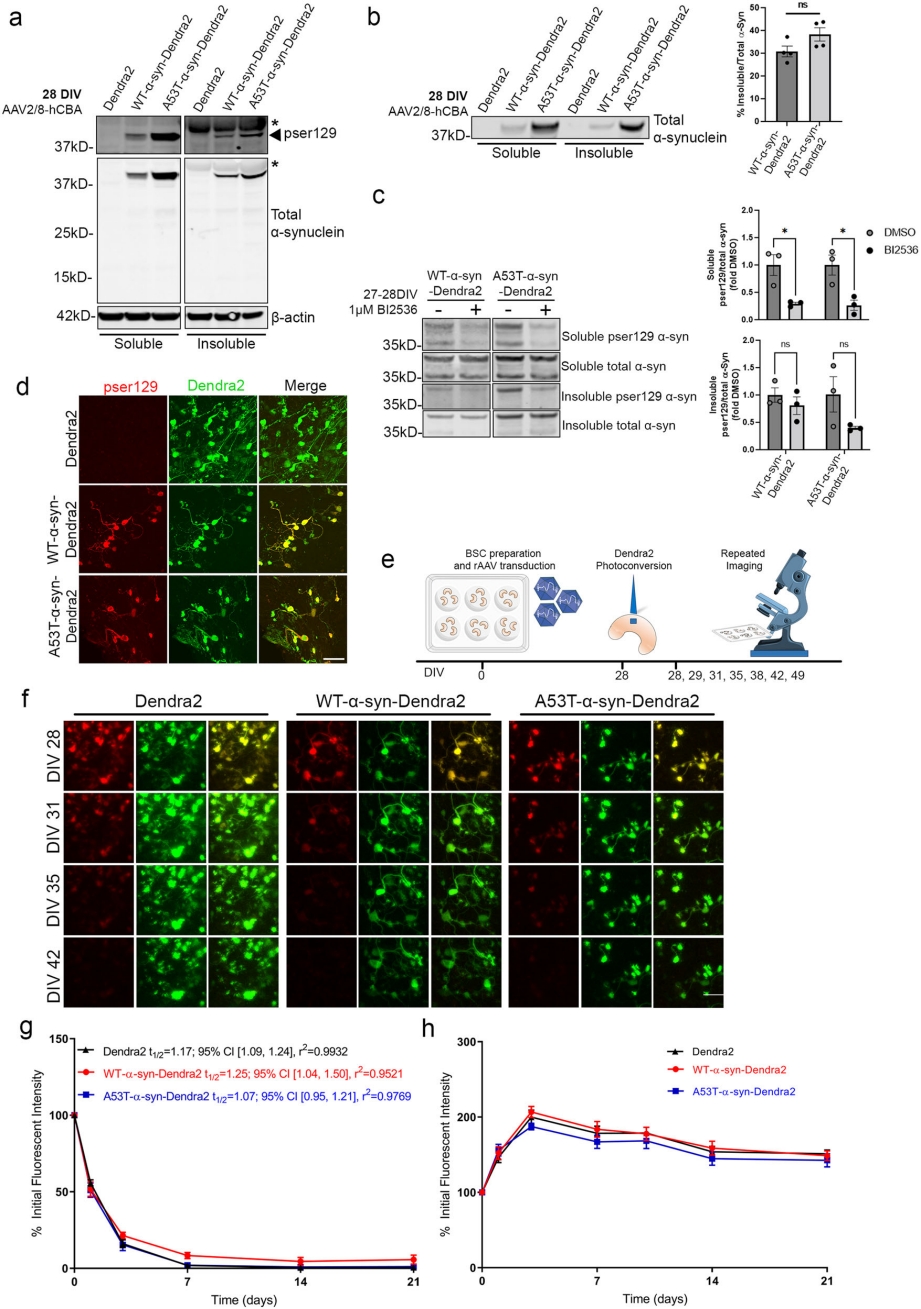
WT and A53T-α-syn-Dendra2 show similar production and turnover rates even when insoluble pSer129 α-syn has accumulated in BSCs. BSCs were transduced at 0 DIV with rAAVs to express Dendra2, WT-α-syn-Dendra2 or A53T-α-syn-Dendra2 and maintained in culture until 28 DIV. **a** BSCs were sequentially extracted to prepare soluble and triton-insoluble fractions. Representative western blots of the soluble and triton-insoluble fractions probed for pSer129 α-syn, total α-syn and β-actin as a loading control are shown. The mobility of molecular mass markers are shown on the left. Black arrow indicates pSer129 (81A), asterisks indicate non-specific bands. (*n* = 3). **b** Equal amounts of soluble and insoluble fractions were loaded onto Western blots and probed for total α-syn. Representative lanes are shown. The mobility of molecular mass markers are shown on the left. The proportion of insoluble α-syn of the total amount was calculated and presented on the bar graph (*n* = 4, mean ± SEM, analyzed by unpaired *T*-test). **c** BSCs were treated with 1 μM BI2536 or DMSO control for 24 h from 27-28 DIV to reduce levels of soluble pSer129. Representative blots of pSer129 α-syn (EP1536Y) and total α-syn are shown. The mobility of molecular mass markers are shown on the left. Bar chart shows levels of soluble pSer129 α-syn are reduced with BI2536 treatment but insoluble pSer129 α-syn is not (*n* = 3, data are mean ± SEM, analyzed by two-way ANOVA with post-hoc Bonferonni’s test). **d** BSCs were fixed, immunostained for pSer129 (EP1536Y) and confocal imaged to confirm the distribution of phosphorylated α-syn. Scale bar = 50 µm. (*n* = 3). **e** Schematic diagram shows the timeline of the long-term optical pulse labeling experiments using photoconversion of Dendra2, WT-α-syn-Dendra2 and A53T-α-syn-Dendra2 at 28 DIV when insoluble, phosphorylated α-syn has accumulated. **f** Representative images of photoconverted (red) and newly synthesized (green) Dendra2 above residual fluorescence in BSCs photoconverted at 28 DIV and imaged at several time points until 49 DIV. Merge of both channels is also shown. Scale bar = 50 µm **g** Line graph shows quantification of population levels of photoconverted Dendra2 fluorescent intensity in each condition that were quantified over time as a proportion of initial fluorescent intensity (*n* = 6, data are mean ± SEM, analyzed by Two-way ANOVA with post-hoc Sidak’s test). **h** Line graphs show quantification of population levels of green Dendra2 fluorescent intensity in each condition that were quantified over time as a proportion of initial fluorescent intensity (*n* = 6, data are mean ± SEM, analyzed by Two-way ANOVA with post-hoc Sidak’s test).

Insoluble, phosphorylated α-syn accumulates in BSCs transduced with α-syn or α-syn-YFP by 28 DIV^15^. Hence, we performed biochemical and immunohistochemical analysis to identify if BSCs transduced with α-syn with a Dendra2 tag also show the development of phosphorylated and insoluble α-syn by 28 DIV. At 28 DIV, overexpression of human α-syn was confirmed in BSCs expressing WT-α-syn-Dendra2 and A53T-α-syn-Dendra2 (Fig. 2a). At this time point, the accumulation of insoluble pSer129 α-syn was also confirmed. We also confirmed no increase in 14 kDa α-syn which may occur if the Dendra2 tag was being cleaved from α-syn (Fig. 2a). We also quantified the proportion of insoluble α-syn at 28 DIV and found that 30.76% ± 4.74% was insoluble in WT-α-syn-Dendra2 BSCs and 38.29% ± 5.90% was insoluble in A53T-α-syn-Dendra2 transduced BSCs (Fig. 2B). The addition of a fluorescent tag such as Dendra2 may influence aggregation kinetics as reported previously^23,24^. To examine this, we also assessed the proportion of insoluble α-syn in BSCs when WT-α-syn and A53T-α-syn were expressed without a tag, and found similar proportions of insoluble α-syn as when a Dendra2 tag is present (Fig. S1A). As at 10DIV, we also applied the PLK1–3 inhibitor BI2536, to improve the detection sensitivity of soluble and insoluble pSer129 and found that only soluble pSer129 was significantly reduced by this treatment in WT-α-syn-Dendra2 and A53T-α-syn-Dendra2 transduced BSCs at 28DIV (Fig. 2c). Distribution of pSer129 α-syn was also assessed with some LB-like features present in WT-α-syn-Dendra2 and A53T-α-syn-Dendra2 transduced BSCs at 28DIV (Fig. 2d).

This characterization confirms that insoluble, phosphorylated α-syn accumulates in BSCs transduced with α-syn with a Dendra2 tag by 28 DIV thereby facilitating the examination of the kinetics of insoluble pSer129 α-syn in subsequent OPL studies. To our knowledge, any estimates of the rate of turnover of α-syn have been performed only in models where the α-syn was predominantly soluble^20,21^ and aggregation and fibrillization may affect α-syn turnover. To take advantage of the rapid development of insoluble, phosphorylated α-syn in our BSC model by 28 DIV; long-term live imaging of α-syn turnover was performed from 28 to 49 DIV (schematic diagram of the long-term imaging timeline in Fig. 2e). Long-term live microscopy from 28 to 49 DIV was used to image the red fluorescent photoconverted Dendra2-tagged α-syn and newly produced non-photoconverted green fluorescent Dendra-2 tagged α-syn (Fig. 2f). Population level fluorescent density quantification revealed no differences in amounts of red photoconverted WT and A53T α-syn even when an abundance of α-syn is insoluble and phosphorylated at this stage (Fig. 2g). When estimating half-lives from this data; Dendra2 alone shows a half-life of 1.17 days; 95% CI [1.09, 1.24], r^2^ = 0.9932, WT-α-syn a half-life of 1.25 days; 95% CI [1.04, 1.50], r^2^ = 0.9521 and A53T-α-syn has an estimated half-life of 1.07 days; 95% CI [0.95, 1.21], r^2^ = 0.9769. These findings suggest that insoluble WT and A53T α-syn turn over at a similar rate as their soluble counterparts and the Dendra2 control. Furthermore, by quantifying the total green fluorescence emitted after photoconversion, we established the presence of insoluble α-syn did not affect α-syn production rates (Fig. 2h). Dendra2 alone, and insoluble WT and A53T α-syn all increased at a similar rate until stabilization.

Overall, by characterizing and then using OPL methods in BSCs we have identified that overexpression, phosphorylation status, insolubility or the A53T variant do not affect the rate of turnover of α-syn in BSC models with LB-like features.

### Seeding of α-syn BSCs with PFFs induces the development of p62 and Thiazin Red positive dystrophic neurons

As with several other neurodegenerative proteinopathies, α-syn pathology progresses spatiotemporally through the brain after disease onset and is believed to be mediated by the seeding and propagation of α-syn species as demonstrated in several cellular and animal models^14,25^.

**Fig. 3:**
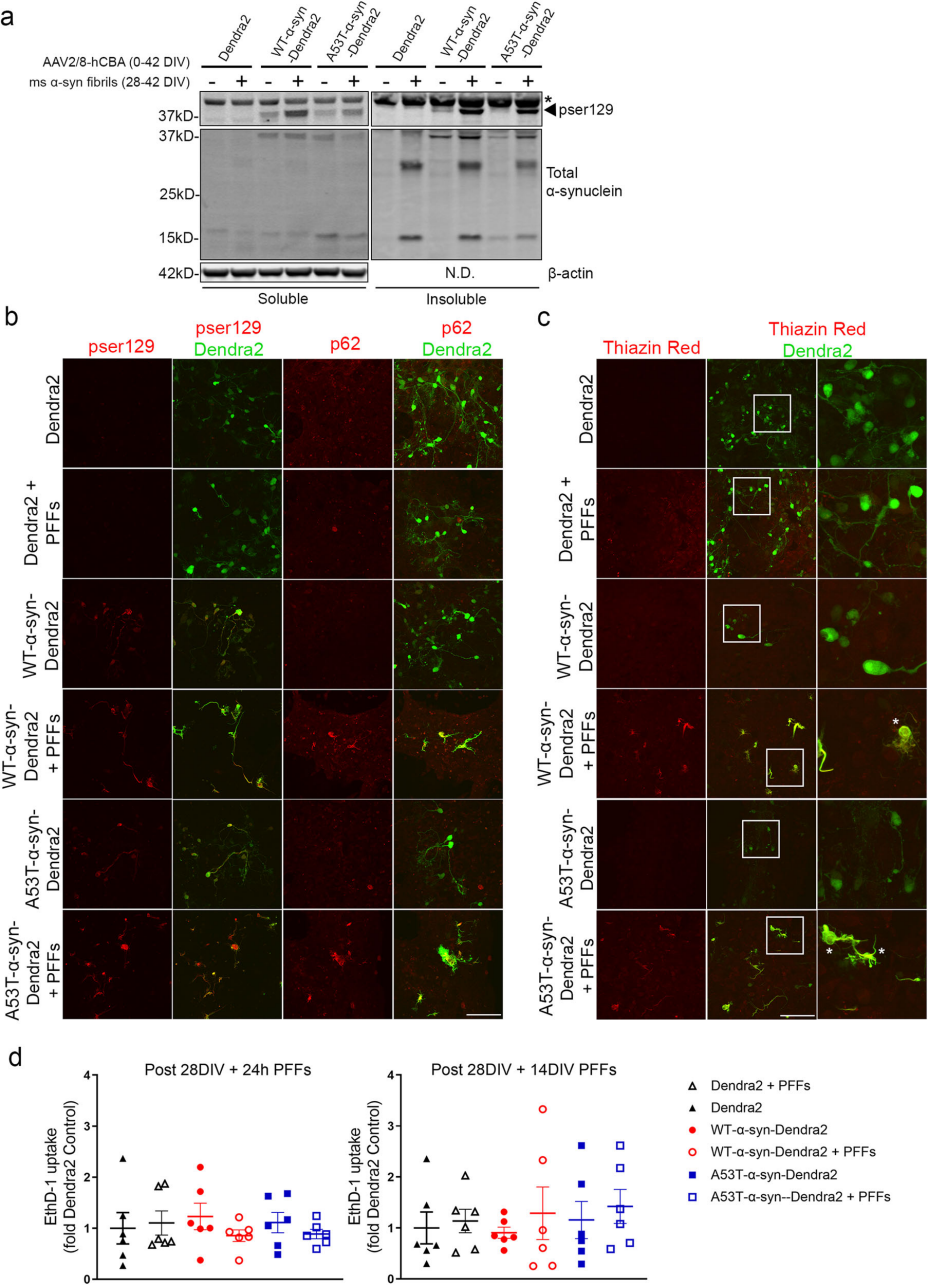
Addition of exogenous mouse α-syn PFFs to WT and A53T-α-syn-Dendra2 transduced BSCs induces p62 immunoreactivity and Thiazin Red accumulation in the absence of toxicity. BSCs were transduced at 0 DIV with rAAVs to express Dendra2, WT-α-syn-Dendra2 or A53T-α-syn-Dendra2, at 28 DIV mouse α-syn PFFs were added and BSCs were harvested or fixed at 42 DIV. **a** BSCs were sequentially extracted to prepare soluble and triton-insoluble fractions. Representative western blots of the soluble and triton-insoluble fractions probed for pSer129 α-syn (EP1536Y), total α-syn and β-actin as a loading control are shown. The mobility of molecular mass markers are shown on the left. Black arrow indicates pSer129, asterisks indicate non-specific bands. (*n* = 3). **b** BSCs were fixed, immunostained for pSer129 α-syn (EP1536Y) or p62 and confocal imaged. Scale bar = 100 µm. (*n* = 2). **c** BSCs were fixed, stained with Thiazin Red and confocal imaged. Scale bar = 100 µm. (*n* = 2). Enlargement is shown and dystrophic processes are marked with white asterisks. **d** To assess any acute or chronic toxicity of α-syn overexpression or PFF addition EthD-1 uptake was assessed 24 h and 14 DIV post-PFF application (*n* = 6, data are mean ± SEM, analyzed by one-way ANOVA).

In addition, some previous studies using BSCs have demonstrated that α-syn PFFs can trigger the formation of LB-like pathology^26–29^. To examine how the addition of exogenous mouse α-syn PFFs affected the biochemical and immunohistochemical profile of α-syn in BSCs we expressed Dendra2, WT-α-syn-Dendra2 and A53T-α-syn-Dendra2 in BSCs with and without the addition of PFFs. Biochemical analysis revealed that BSCs expressing both WT and A53T human α-syn accumulate insoluble and pSer129 α-syn with the PFFs also being detected (Fig. 3a). We did not identify any seeding of endogenous mouse α-syn in BSCs expressing Dendra2 alone (Fig. 3a). To further examine the LB-like pathology in the presence of α-syn PFFs we immunohistochemically examined the presence of pSer129 and p62 in BSCs, as p62 is often found in LBs in human α-Synucleinopathies^30^. Both WT and A53T α-syn transduced BSCs with and without PFFs addition showed pSer129 positivity, but that is increased with PFFs (Fig. 3b).

Although the widely accepted and predominant marker of LB pathology is pSer129 α-syn^5,6^ it is becoming more established that LBs are highly complex structures comprised of α-syn and several other proteins and lipid species which may link more tightly with neurodegeneration^8,10,31^. As p62 is commonly abundant in LBs in human α-Synucleinopathies^30^ we examined whether p62 was also present in these inclusions. Only WT and A53T α-syn transduced BSCs with the addition of PFFs showed p62 positivity and only in a small proportion of cells (Fig. 3b), suggesting that the addition of PFFs adds a new level of complexity to the pathology in some human α-syn expressing cells. To further detect fibrillar structures in α-syn-Dendra2 transduced BSCs we used the dye Thiazin Red^17^. Thiazin Red staining shows some heterogeneity in LB detection and appears to principally identify later stage Lewy bodies in human PD^32^. Only BSCs transduced with WT and A53T α-syn with the addition of PFFs showed detectable Thiazin Red positivity and similar to p62, only in a small proportion of cells (Fig. 3c), with staining absent in BSCs transduced with Dendra2 or without PFFs, suggesting the detection of structural features common to later stage Lewy bodies in human PD. Notably, there appeared to be some strong morphological changes in Thiazin Red and p62 positive cells where WT and A53T α-syn were expressed in the presence of PFFs, with cells appearing to look largely dystrophic in nature with swollen processes (enlargement Fig. 3c).

The application of PFFs in BSC models at high doses can cause neurotoxicity^29^. To examine any toxicity here due to PFF application we assessed EthD-1 uptake in the BSCs, acutely (24 h) and chronically (14 DIV) post application (Fig. 3d). No significant toxicity of the rAAV α-syn overexpression or PFF application was detected.

As the rAAV promoters used here can potentially transduce cells other than neurons we examined Thiazin Red positivity in BSCs using rAAVs that express α-syn only in neurons and without the Dendra2 tag. We identified that when untagged WT-α-syn and A53T-α-syn are expressed in BSCs and seeded with PFFs, Thiazin Red accumulation is also triggered in the PFF conditions as is observed with a Dendra2 tag (Fig. S2A). Additionally, when WT-α-syn and A53T-α-syn are driven to express in neurons using the Synapsin promoter and PFFs added, Thiazin Red accumulates in a proportion of α-syn-Dendra2 neurons (Fig. S2B) which also appear swollen and dystrophic. Furthermore, we performed immunohistochemistry of cell-specific markers to identify which cells were mainly transduced with the rAAVs (Fig. S2C). WT-α-syn-Dendra2 and A53T-α-syn-Dendra2 expression with or without PFF application was mainly detected in neurons.

Taken together, these findings reveal that the addition of PFFs to neural cells overexpressing α-syn induces changes which are not seen without PFFs. Future studies should identify whether this is akin to a more mature LB phenotype.

### Exogenous α-syn PFFs slow α-syn-Dendra2 protein turnover in α-syn overexpressing BSCs

The triggering of α-syn pathology by the addition or injection of PFFs to cell or animal models has been a common strategy to model aspects of LB pathology^14,25,33^ but the precise dynamics of α-syn inclusions induced by seeding remain to be elucidated.

**Fig. 4:**
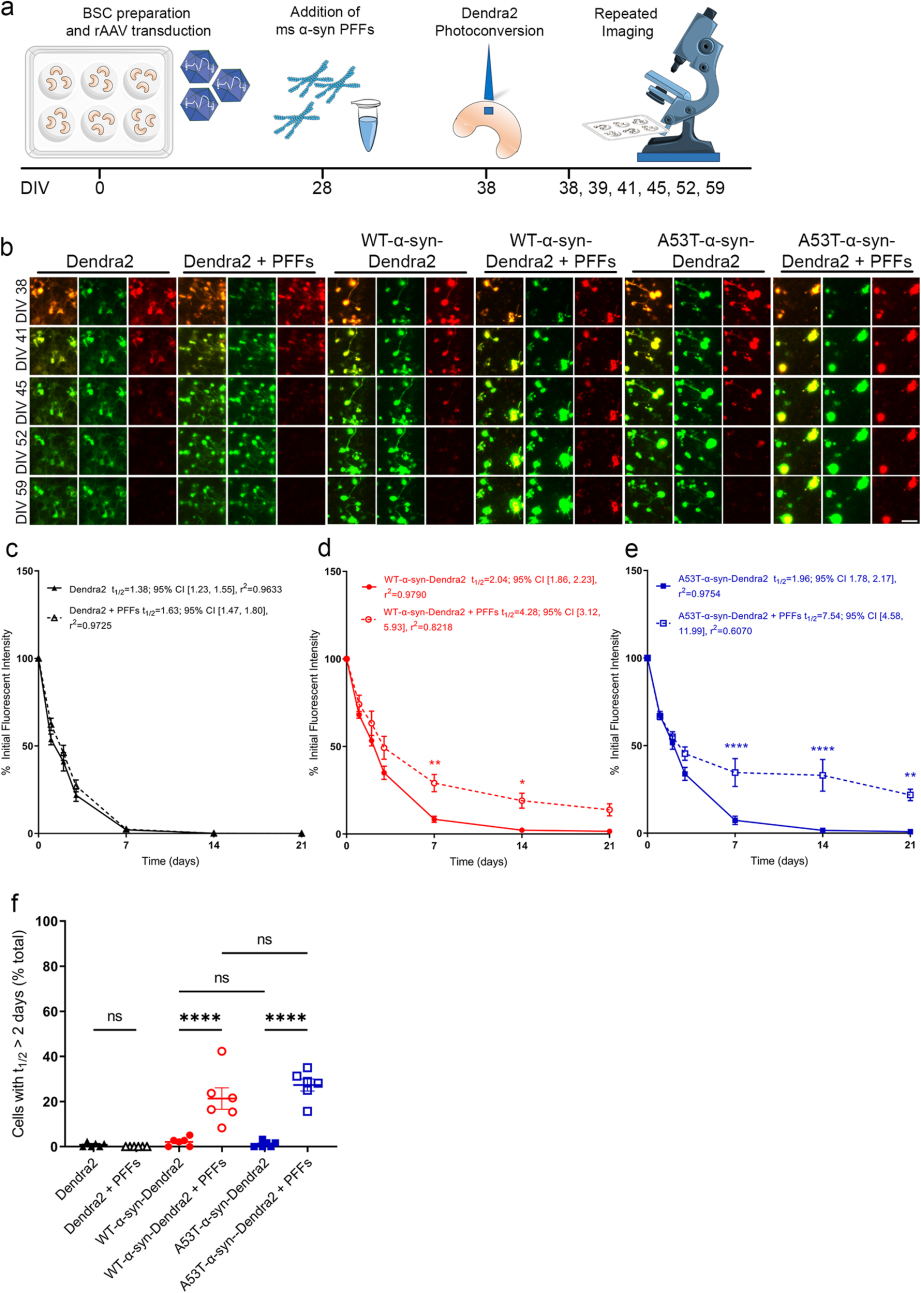
Addition of mouse α-syn PFFs slows WT and A53T-α-syn-Dendra2 turnover and is attributed to a proportion of cells that become unable to degrade α-syn. BSCs were transduced at 0 DIV with rAAVs to express Dendra2, WT-α-syn-Dendra2 or A53T-α-syn-Dendra2, at 28 DIV mouse α-syn PFFs were added and optical pulse labeling experiments began at 38 DIV. **a** Schematic diagram shows the timeline of the long-term optical pulse labeling experiments using photoconversion at 38 DIV of Dendra2, WT-α-syn-Dendra2 and A53T-α-syn-Dendra2 after mouse α-syn PFFs application at 28 DIV. **b** Representative images of photoconverted (red) and newly synthesized (green) Dendra2 above residual fluorescence in BSCs. Merge of both channels is also shown. Scale bar = 50 µm **c**–**e** Line graphs show quantification of population levels of red photoconverted Dendra2 fluorescent intensity in each condition that were quantified over time as a proportion of initial fluorescent intensity (*n* = 6, data are mean ± SEM, analyzed by Two-way ANOVA with post-hoc Sidak’s test, ^*^p < 0.05 ^**^p < 0.01 ^****^p < 0.0001). **f** Graphs show estimated % of cells that show an average half-life greater than 2 days which contributes to the lengthening of the population half-life (*n* = 6, data are mean ± SEM, analyzed by One-way ANOVA with Tukey’s multiple comparisons test, ^****^p < 0.0001, ns not significant).

After observing that PFFs induce WT-α-syn-Dendra2 and A53T-α-syn-Dendra2 to form more complex LB pathology that is p62 immunoreactive and Thiazin Red positive 10–14 days after addition, we aimed to compare the longevity of WT-α-syn-Dendra2 and A53T-α-syn-Dendra2 in the presence and absence of PFFs using OPL. A schematic diagram of the imaging timeline is shown in Fig. 4a. Long-term live microscopy from 28 to 49 DIV was used to image the red fluorescent photoconverted Dendra2-tagged α-syn and newly produced non-photoconverted green fluorescent Dendra2-tagged α-syn±PFFs (Fig. 4b). Population level fluorescent density quantification revealed no differences in amounts of red photoconverted Dendra2 ± PFFs (Fig. 4c); Dendra2 alone shows an estimated half-life of 1.38 days; 95% CI [1.23, 1.55], r^2^ = 0.9633, and Dendra2 + PFFs has an estimated half-life of 1.63 days; 95% CI [1.47, 1.80], r^2^ = 0.9725. Population level red fluorescent density quantification revealed the addition of PFFs to WT-α-syn-Dendra2 significantly slowed α-syn turnover (Fig. 4d); WT-α-syn-Dendra2 alone shows an estimated half-life of 2.04 days; 95% CI [1.86, 2.23], r^2^ = 0.9790, and WT-α-syn-Dendra2 + PFFs has an estimated half-life of 4.28 days; 95% CI [3.12, 5.93], r^2^ = 0.8218. The addition of PFFs to A53T-α-syn-Dendra2 also significantly slowed α-syn turnover (Fig. 4e); A53T-α-syn-Dendra2 alone shows an estimated half-life of 1.96 days; 95% CI [1.78, 2.17], r^2^ = 0.9754, and A53T-α-syn-Dendra2 + PFFs has an estimated half-life of 7.54 days; 95% CI [4.58, 11.99], r^2^ = 0.6070. As population level analysis revealed the slowing of both WT-α-syn-Dendra2 and A53T-α-syn-Dendra2 in the presence of PFFs, we calculated the proportion of cells which contributed to the slowing of turnover by quantifying the proportion of cells that showed a half-life of greater than the control average (2 days). We identified that 21.30 ± 4.74% of WT-α-syn-Dendra2 + PFFs cells showed a significant slowing of α-syn turnover compared to 2.12 ± 0.79% of WT-α-syn-Dendra2 cells. Similarly, 27.33 ± 2.70% of A53T-α-syn-Dendra2 + PFFs cells showed a significantly increased slowing of α-syn turnover compared to 1.092 ± 0.54% of A53T-α-syn-Dendra2 cells. Furthermore, to examine whether this slowing of turnover and degradation is occurring in α-syn bearing neurons, we tracked WT-α-syn-Dendra2 and A53T-α-syn-Dendra2 with expression mediated with the Synapsin promoter in the presence and absence of PFFs using OPL according to the timeline in Fig. S3A. It was observed again that a proportion of neurons show slowing of turnover and persistence of red fluorescence beyond 2 weeks suggesting this slowing of turnover occurs in neurons where a proportion of cells have significantly slowed α-syn clearance (Fig. S3B).

In summary, the addition of PFFs significantly slows the turnover of WT-α-syn-Dendra2 and A53T-α-syn-Dendra2 in neurons and this lengthening of half-life is driven by approximately 25% of cells where α-syn clearance is significantly slowed. Future studies should ascertain the mechanisms behind this slowing of α-syn turnover and potential degradation deficits. This platform provides a flexible tool to provide mechanistic insight and to identify whether this can be therapeutically modulated.

## Discussion

Using long-term OPL in BSCs expressing WT-α-syn-Dendra2 and A53T-α-syn-Dendra2 we identify that upon their initial accumulation, insoluble or pSer129 α-syn do not alter α-syn protein dynamics. In addition, the PD-linked A53T mutation and overexpression of human α-syn do not affect α-syn clearance or production. Only upon addition of exogenous α-syn PFFs to α-syn-overexpressing BSCs is clearance of α-syn slowed, and that is attributed to approximately 25% of cells which retain photoconverted α-syn, become p62 immunoreactive and Thiazin Red positive and adopt a dystrophic morphology. Our data suggests that, at least in conditions without seeding, and even in most cells where the application of PFFs has not stabilized the overexpressed α-syn-Dendra2 then α-syn continues to turnover in a cell and this is continually replaced with newly synthesized α-syn.

pSer129 α-syn is a classical identifier of LBs and LNs used across pathological and modeling studies to distinguish LB pathology^5,6,9,14^. In these studies, we demonstrate that even when soluble or insoluble pSer129 α-syn accumulate in WT-α-syn-Dendra2 and A53T-α-syn-Dendra2 BSCs, turnover of α-syn occurs at similar rates to Dendra2 alone. This highlights that phosphorylation or the accumulation of insoluble α-syn does not immediately affect α-syn turnover, particularly when this α-syn has initially accumulated. Furthermore, by using the rAAV constructs we drive overexpression of human α-syn and again this does not affect α-syn protein dynamics in this system. This is in line with a previous study where human α-syn-Dendra2 overexpression in rat primary neurons induced soluble pSer129 α-syn accumulation but did not affect α-syn turnover rates^20^. These new data extend these findings by showing that the accumulation of insoluble pSer129 α-syn at least upon initial accumulation does not affect α-syn clearance or production, and also in an intact system comprised of all CNS cell types which could modulate α-syn clearance mechanisms. This is important, as other studies have suggested that pSer129 may encourage degradation by autophagy or the proteasome^34–36^ and it appears these mechanisms may be operating functionally here, but also may reach capacity particularly in the context of PFFs or if a threshold of pSer129 is reached. Furthermore, the finding that neurons bearing insoluble pSer129 α-syn maintain regular α-syn turnover rates suggests that cells can compensate, likely remain functional at this stage and that interventions targeting α-syn^2^ may be possible at this stage. Similarly, the initial accumulation of insoluble pSer129 α-syn is not disruptive to α-syn degradation even in the context of α-syn overexpression.

In these studies, we identified that A53T-α-syn-Dendra2 did not affect turnover, suggesting this PD-linked mutation’s contribution to disease does not appear to affect the stability or degradation of α-syn. This is in contrast with previous data in neurons or cell lines suggesting A53T extends the half-life and stability of α-syn^21,22,37,38^ but is in agreement with other studies suggesting that the A53T variant does not extend half-life and neither does A30P or E46K^20,39^. It is important to note that using OPL in BSCs provides an intact system to study α-syn turnover with relevant CNS cell types and without the need of inhibitors^40^ which may confound or show point mutation specific effects in clearance.

Our studies estimate the population half-life of α-syn to be 1-2 days in agreement with previous studies using human α-syn overexpression^39,41^ or endogenous mouse α-syn^37^. This was the case even when we observed soluble and insoluble pSer129 accumulation. These half-life estimates, however, were dramatically extended upon the addition of PFFs. Upon PFF addition, we observed the accumulation of p62 and Thiazin Red positivity as well as pSer129 positivity in α-syn-Dendra2 BSCs. This in turn resulted in a slowing of α-syn turnover in approximately 25% of cells. This suggests that this extra layer of α-syn pathology induction, may represent later stages of disease or even different inclusion formation. It also may be suggestive of a window where pathology is more LB-like rather than mature LBs and can be explored further in this system. This adds to the emerging data that a true LB is more than α-syn accumulation^10,42^. This data complements previous in vivo mouse multiphoton imaging studies which show that α-syn turnover slows in PFF-induced inclusions but estimated half-lives were not quantified^43^.

Of note, expression of Dendra2 alone with PFFs did not show pSer129 accumulation in contrast to previous reports that PFFs can seed endogenous mouse α-syn in BSCs^26–29^. This is likely due to different application methods of PFFs, brain regions, timings, culture conditions, as well as different PFF concentrations which likely contribute to why this was not observed in our paradigm.

Equally, A53T α-syn did not seem more amenable to seeding than WT human α-syn, with both showing slowed α-syn turnover rates and a similar proportion of cells unable to degrade α-syn at normal rates.

Despite their discovery over 100 years ago, there is still limited consensus on the effects and contributions of LBs and chiefly α-syn as a component of LBs to disease progression in PD and LBD^10^. In particular, there has been a heavy focus on the accumulation and aggregation of α-syn as a disease driver, particularly since the identification of *SNCA* being a causal PD gene^1^. More recently, the complex nature of LBs has emerged, with membranes, vesicles, organelles, lipids and an abundance of other proteins being detected in modeled LBs and LBs from end-stage post-mortem PD brain, with non-fibrillar forms of α-syn being the main α-syn species found in LBs^8,10,11,42,44^. Furthermore, it is evident that LBs are likely to be a heterogenous population comprised of different components^10^. Here as we show that inclusions that bear insoluble pSer129 α-syn accumulation did not have diminished α-syn turnover, this further adds to the evidence that aggregated α-syn is necessary but may not be sufficient for mature LB formation and that this may represent an early disease stage^31^. Also, it appears that this system will be a useful tool in future studies assessing this disease aspect and characterizing LB formation and progression.

Our finding that insoluble α-syn and pSer129 α-syn accumulation and overexpression do not affect α-syn turnover suggests that it is likely traditional α-syn clearance mechanisms continue to facilitate normal degradation in these conditions^34^. Future studies should address the precise mechanisms by which these processes occur and any other factors which may halt this normal degradation (beyond the addition of PFFs, p62 accumulation and Thiazin Red positivity). It will also be important to determine how long these typical α-syn clearance processes are sustainable for, as this will influence how we should therapeutically target these inclusions in disease. Similarly, the use of tagged α-syn-Dendra2 here may have slightly different aggregation kinetics to untagged α-syn^23^ but where possible to examine we showed features of α-syn-Dendra2 BSCs mimicked those of untagged α-syn BSCs and future studies should account for this. It will also be important to determine whether the cells with slowed α-syn turnover have taken up the PFFs and this is what has caused the slowing – or whether the formation of a more complex LB or different type of inclusion in a proportion of cells is triggered by the contact of PFFs with overexpressed α-syn or alternative mechanisms. Recent studies have shown that seeded primary neuronal cultures take up the PFFs in around 22% of neurons^31^, which is similar to the proportion and timeline studied here, suggesting this may drive the slowed clearance. In the case of α-syn, a wide range of clearance mechanisms have been proposed and experimentally documented including proteasomal or lysosomal degradation, other CNS cell types degrading α-syn and extracellular clearance mechanisms^27,34,45–47^.

It is likely these mechanisms will be highly dependent on the species or any post-translational modifications of α-syn, however these can be further explored in this system. Further, this system can be used to examine how any therapies may affect the turnover of α-syn particularly in the context of PFFs where our data suggests deficits in degradation.

In summary, our findings identify that insoluble or pSer129 α-syn do not affect the rates of α-syn protein dynamics, at least in the initial period when LB-like pathology has accumulated in BSCs. Degradation of α-syn is slowed in approximately 25% of cells when exogenous α-syn PFFs are applied, and this is associated with the accumulation of p62 and Thiazin Red positivity and a dystrophic morphology. It will now be important to dissect the mechanisms underlying this slowing of turnover in the presence of PFFs and its implications for therapies targeting α-syn. This also particularly invites further investigation of the staging of LB formation and their impact on disease. Similarly, it appears that the A53T α-syn mutation alone does not slow α-syn turnover suggesting impaired α-syn clearance may not occur in PD-linked A53T carriers as a sole mechanism of disease progression at least at early disease stages.

In conclusion, these studies have furthered our understanding of effects of different forms of α-syn on protein turnover, and this facile platform enables future investigation of modifiers of α-syn protein dynamics and the mechanisms underlying any slowing of turnover and possible therapeutic interventions.

## Methods

### rAAV production and preparation

rAAV 2/8 expressing Dendra2^48^, human WT α-syn with the C-terminal Dendra2 tag and human A53T α-syn with a C-terminal Dendra2 tag, under the control of the hybrid CBA chicken β-actin promoter with cytomegalovirus enhancer (CMV) enhancer were generated^15,17^. Dendra2, WT-α-syn-Dendra2, A53T-α-syn-Dendra2 were also generated under the control of the human synapsin-1 promoter and packaged in rAAV 2/8-3Y^49^.

### Organotypic brain slice cultures

All mouse procedures were approved by the Institutional Animal Care and Use Committee at the University of Florida or at the Francis Crick Institute in accordance with the UK Animals (Scientific Procedures) Act 1986. BSCs were prepared from postnatal day 8 (p8) B6/C3H F1 mice (Envigo, Indianapolis, IN, USA) or C57BL/6J mice (The Jackson Laboratory, ME, USA)^15,50^. In brief, 350 µm coronal slices containing the cortex or cortex and hippocampus were cut using a McIllwain^TM^ tissue chopper (Mickle Laboratory Engineering Co. Ltd., Surrey, UK) in sterile-filtered Hank’s balanced salt solution (HBSS), calcium, magnesium, no phenol red (Thermo Fisher Scientific), 2 mM ascorbic acid (Sigma Aldrich, St Louis, MO, USA), 39.4 μM ATP (Sigma Aldrich), and 100 units/ml penicillin, 100 µg/ml streptomycin (Thermo Fisher Scientific). Brain slices were collected and plated randomly across wells to avoid any regional effects to contain three slices per semi-porous membrane insert (Millipore, 0.4 µm pore diameter, Thermo Fisher Scientific) in 6-well sterile culture plates. Slices were maintained at 37 °C and 5% CO_2_ in sterile-filtered culture medium (basal medium eagle (BME, Thermo Fisher Scientific), 26.6 mM HEPES (pH 7.1, Thermo Fisher Scientific), 40 mM Glucose (Sigma Aldrich), 511 μM ascorbic acid, 1% (v/v) GlutaMAX (Thermo Fisher Scientific), 0.033% (v/v) insulin (Sigma Aldrich), 100 units/ml penicillin, 100 µg/ml streptomycin (Thermo Fisher Scientific) and 25% (v/v) heat-inactivated horse serum (Sigma Aldrich). Slice culture medium was exchanged every 3-4 days for long-term maintenance. rAAVs were applied directly into the culture medium on the first day in vitro (DIV 0) at 1-2 × 10^10^ VGs of rAAV per well containing 3 BSCs.

### BI2536 treatment of BSCs

BSCs were treated with 1 μM BI2536 (Aobious Inc, MA, USA), or dimethylsulfoxide (DMSO) control added to the media and 1 μL added on top of the slice, for 24 h from 9-10 DIV or 27-28DIV to reduce levels of soluble pSer129 α-syn and identify any insoluble pSer129 α-syn^16^.

### Extraction of BSC lysates

BSCs for assessment of insoluble α-syn from three wells (nine slices in total) were pooled together and harvested into ice-cold PBS and presented as *n* = 1. BSCs were washed and then lysed in 1% Triton X-100 high salt (HS) buffer (50 mM Tris–HCl, pH 7.5, 0.75 M NaCl) containing protease and phosphatase inhibitors. Lysates were then centrifuged at 100,000 *g* for 20 min at 4 °C. Supernatants were removed (Soluble fraction), and the pellet was washed in HS buffer with sucrose and centrifuged at 100,000 *g* for 20 min at 4 °C. The pellet was then resuspended in 2% SDS, sonicated, and heated to 100 °C for 10 min (Triton-insoluble fraction)^15,51^. Protein concentrations were determined by bicinchoninic acid (BCA) assay (Thermo Fisher Scientific) before equal amounts of protein were resolved by immunoblotting. To calculate the proportion of insoluble α-syn, BSCs were extracted in the same manner but equal volumes of soluble and insoluble fractions were resolved by immunoblotting before determining the proportion of insoluble/total α-syn.

### Antibodies

The following antibodies were used for Western blotting and immunohistochemistry. SNL-4, a rabbit polyclonal antibody raised against amino acid sequence 2–12 of human α-syn^52^; 94-3A10, a mouse monoclonal antibody which recognizes amino acid sequence 130–140 of human α-syn^53^; 81A, a mouse monoclonal antibody specific for α-syn phosphorylated at Ser129^54^; and a rabbit monoclonal antibody specific for α-syn phosphorylated at Ser129 (EP1536Y; Abcam) were used. A rabbit polyclonal antibody for p62/SQSTM1 (18420-1-AP; Proteintech Group Inc, Rosemont, IL, USA) was also used. A mouse monoclonal antibody to β-actin (A5441; Sigma Aldrich), was also used. NeuN was detected using a mouse monoclonal antibody (MAB377; Millipore); GFAP detected with a rabbit polyclonal antibody (Z0334; Agilent, Santa Clara, CA, USA); Iba1 detected with a rabbit polyclonal antibody (234003, Synaptic Systems GmBH, Goettingen, Germany) and Olig2 detected with a rabbit polyclonal antibody (AB9610; Millipore).

### SDS-PAGE and western blotting

15–25 µg protein was separated on 10% (w/v) SDS-PAGE gels (Bio-Rad Laboratories, Hercules, CA, USA) and electrophoretically transferred to polyvinylidene difluoride (PVDF) membranes^15,55^. Membranes were blocked in 0.5% casein for 1 h and then incubated with primary antibodies overnight at 4 °C. For BI2536/DMSO-treated samples and samples where insoluble/soluble proportions were calculated, proteins were separated on 4–12% SDS-PAGE gels (Thermo Fisher Scientific) and transferred to nitrocellulose membranes with 5% milk as block. After primary antibody incubation, membranes were washed 3 times with Tris-buffered saline (TBS) before incubation with fluorophore-conjugated Alexa Fluor 680 anti-mouse IgG (Thermo Fisher Scientific) or IRDye 800 goat anti-rabbit IgG (Li-Cor Biosciences, Lincoln, NE, USA) secondary antibodies for 1 h and washed 3 times with TBS. Protein bands were detected using the multiplex Li-Cor Odyssey Infrared Imaging system (Li-Cor Biosciences).

### Immunohistochemistry and confocal imaging

BSCs were washed in PBS and then fixed on their inserts in 4% paraformaldehyde (PFA) for 1 h^56^. Individual BSCs (*n* = 1) were cut out from their membranes after fixation and then used as free-floating sections for subsequent steps. BSCs were permeabilized and blocked for 18 h in 0.5% Triton X-100 in 20% bovine serum albumin (BSA) (Sigma Aldrich) at 4 °C. BSCs were then incubated overnight at 4 °C with the appropriate primary antibodies in 5% BSA, washed and then incubated with fluorophore-coupled secondary antibodies for 4 h at RT. Slice cultures were washed a further three times before mounting on slides with Fluoromount-G (Southern Biotech, Birmingham, AL, USA) and then imaged using an Olympus FV1200 IX83 confocal laser-scanning microscope (Olympus America Inc, Center Valley, PA, USA) or Zeiss LSM880 confocal laser-scanning microscope (Carl Zeiss AG, Oberkochen, Germany). Z-stacks were captured at recommended step-sizes and projected as a maximum projection image using the Olympus Fluoview FV10-ASW Version 4.02 or Zeiss Zen Blue 3.4 software.

### Long-term live imaging and quantification of Dendra2 in BSCs

Images of living BSCs were captured using a Keyence BZ-X700 all-in-one fluorescence microscope (Keyence Corporation of America, Itasca, IL, USA). Dendra2 transduced BSCs were photoconverted using a 2 s pulse of blue light through a 20 µm Z-stack at recommended step-sizes with a ×20 objective lens. Images of emitted green and red fluorescence through a 20 µm Z-stack at recommended step-sizes were captured at the relevant time points and then projected onto a full focus image using the BZ-analyzer version 1.3.1.1 software (Keyence Corporation of America). Each red and green fluorescent image from each slice at each time point was captured from pre-determined regions of the cortex of the BSCs for each group, and BSCs analyzed included rostral through caudal cortical regions for each group. For population analysis, total corrected fluorescence was calculated using integrated density above background integrated density after thresholding for each BSC imaging series using ImageJ (Version 1.51 k, National Institutes of Health, Bethesda, MD, USA). For quantification of the cells that showed a lengthened half-life, the number of red cells as a proportion of green cells at DIV 14 was calculated after thresholding.

### Expression, purification, assembly, and addition of WT full-length mouse α-syn fibrils

Recombinant full length mouse α-syn was expressed from pRK172 plasmids containing the cDNA for the *Snca* gene and expressed in E.coli BL21 (DE3). Recombinant protein was purified utilizing size exclusion and Mono Q anion exchange chromatography^57,58^. Proteins were diluted in pH 7.4 sterile PBS, and concentrations were determined by BCA assay. Recombinant mouse α-syn protein was assembled into fibrils by incubation at 37 °C at 5 mg/ml in sterile PBS with continuous shaking and fibril assembly was monitored with K114 fluorometry^57,58^. Mouse α-syn fibrils were diluted in sterile PBS and underwent bath sonication for 1 h at RT. In experiments where BSCs were seeded, 2 μg of mouse α-syn PFFs were applied directly on top of each BSC at the times indicated in schematic diagrams (Figs. 4a, S3A).

### Thiazin Red staining

BSCs were washed in PBS and then fixed on their inserts with 4% PFA for 1 h. BSCs were stained with 0.05% Thiazin Red^17^. Individual BSCs (*n* = 1) were cut from their membranes and autofluorescence reagent (Millipore) was applied for 5 min, and then washed in 40% ethanol (EtOH). BSCs were incubated with 0.05% Thiazin Red in ddH_2_O for 3 min in the dark, and then washed in 50% EtOH, PBS and then ddH_2_O. BSCs were mounted on slides with Fluoromount-G (Southern Biotech) and then confocal imaged to identify any amyloidogenic β-sheet structures in these sections.

### Ethidium homodimer-1 (EthD-1) uptake toxicity assay

EthD-1 uptake was used to assess cytotoxicity^15^. BSCs were washed and then incubated with 4 µM EthD-1 in PBS for 15 min and then three images of each slice were captured blindly from predetermined regions using a ×10 objective lens. Positive cells were counted using the analyze particles feature on ImageJ and the mean number of positive cells calculated from the three images captured.

### Statistical analysis

Data were analyzed statistically according to the methods specifically referred to in each figure legend. Data were compared by Student’s two-tailed unpaired *T*-test, One-way analysis of variance (ANOVA) with post-hoc Tukey’s multiple comparisons test or Two-way ANOVA with post-hoc Sidak’s test (Graphpad Prism 9.0 Software, La Jolla, CA, USA). Half-lives were calculated using non-linear regression analysis of one-phase decay. Differences were considered statistically significant when adjusted *p* < 0.05. All graphs were generated in GraphPad Prism.

## Supplementary information

Supplementary Information

## Data Availability

The datasets generated during and/or analyzed during the current study are available from the corresponding authors on request.
