# Supplementary material for: Optical pulse labeling studies reveal exogenous seeding slows α-synuclein clearance
Source: NPJ Parkinsons Dis. 2022 Dec 19;8:173. doi: 10.1038/s41531-022-00434-4 (PMC9763367; doi:10.1038/s41531-022-00434-4)

Croft et al. Optical pulse labeling studies reveal exogenous seeding slows α-synuclein clearance
Supplementary Information:

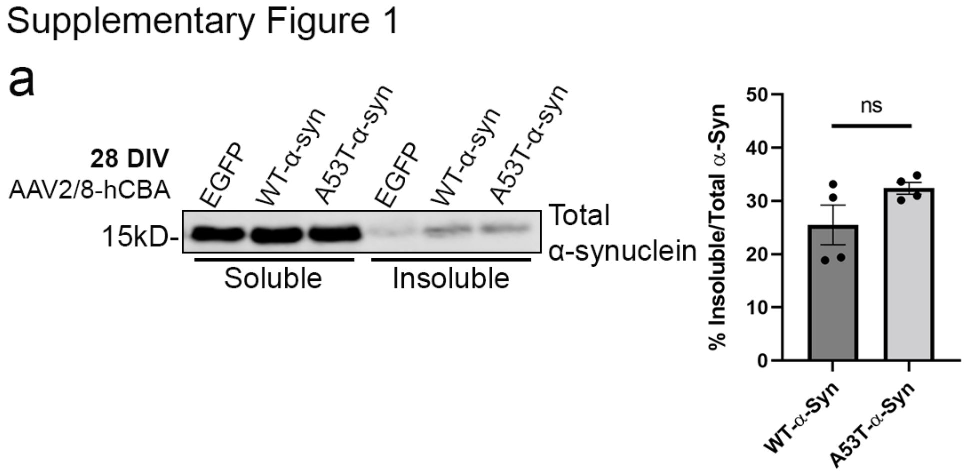

Supp. Figure 1: WT and A53T-α-syn BSCs accumulate insoluble α-syn at 28 DIV (A) BSCs were transduced at 0 DIV with rAAVs to express EGFP, WT-α-syn or A53T-α-syn. At 28 DIV, BSCs were sequentially extracted to prepare soluble and Triton-insoluble fractions. Equal amounts of soluble and Triton-insoluble fractions were loaded onto Western blots and probed for total α-syn. Representative lanes are shown. The mobility of molecular mass markers are shown on the left. The proportion of insoluble α-syn of the total amount was calculated and presented on the bar graph (n=4, mean ± SEM, analyzed by unpaired T-test).

Supplementary Figure 2

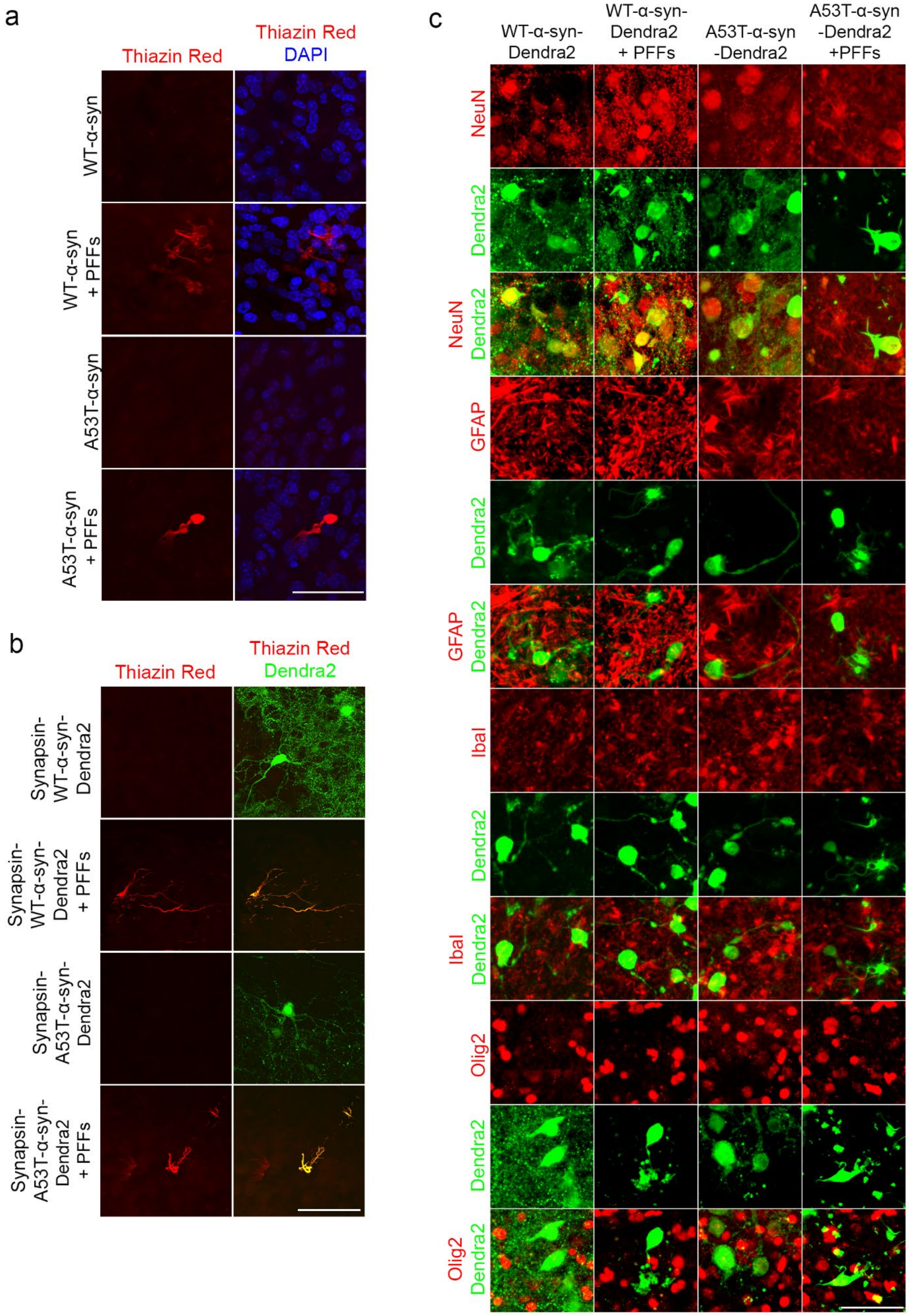

Supp. Figure 2: Thiazin Red accumulates in the presence of exogenous mouse α-syn PFFs and rAAVs mainly express in neurons

(A) BSCs were transduced at 0 DIV with rAAVs to express WT-α-syn or A53T-α-syn, at 28 DIV mouse α-syn PFFs were added and BSCs were fixed, stained with Thiazin Red and confocal imaged 14 days later. Scale bar = 50 μm. (n=3). (B) BSCs were transduced at 0 DIV with rAAVs to express WT-α-syn-Dendra2 or A53T-α-syn-Dendra2 under the Synapsin promoter to drive expression to neurons, at 28 DIV mouse α-syn PFFs were added and BSCs were fixed, stained with Thiazin Red and confocal imaged 14 days later. Scale bar = 50 μm. (n=3). (C) BSCs were transduced at 0 DIV with rAAVs to express WT-α-syn-Dendra2 or A53T-α-syn-Dendra2, at 28 DIV mouse α-syn PFFs were added for a further 14 DIV. BSCs were then fixed, stained for NeuN, GFAP, IbaI, and Olig2 to identify neurons, astrocytes, microglia and oligodendrocytes, respectively and confocal imaged. Scale bar = 50 μm. (n=3).

Supplementary Figure 3

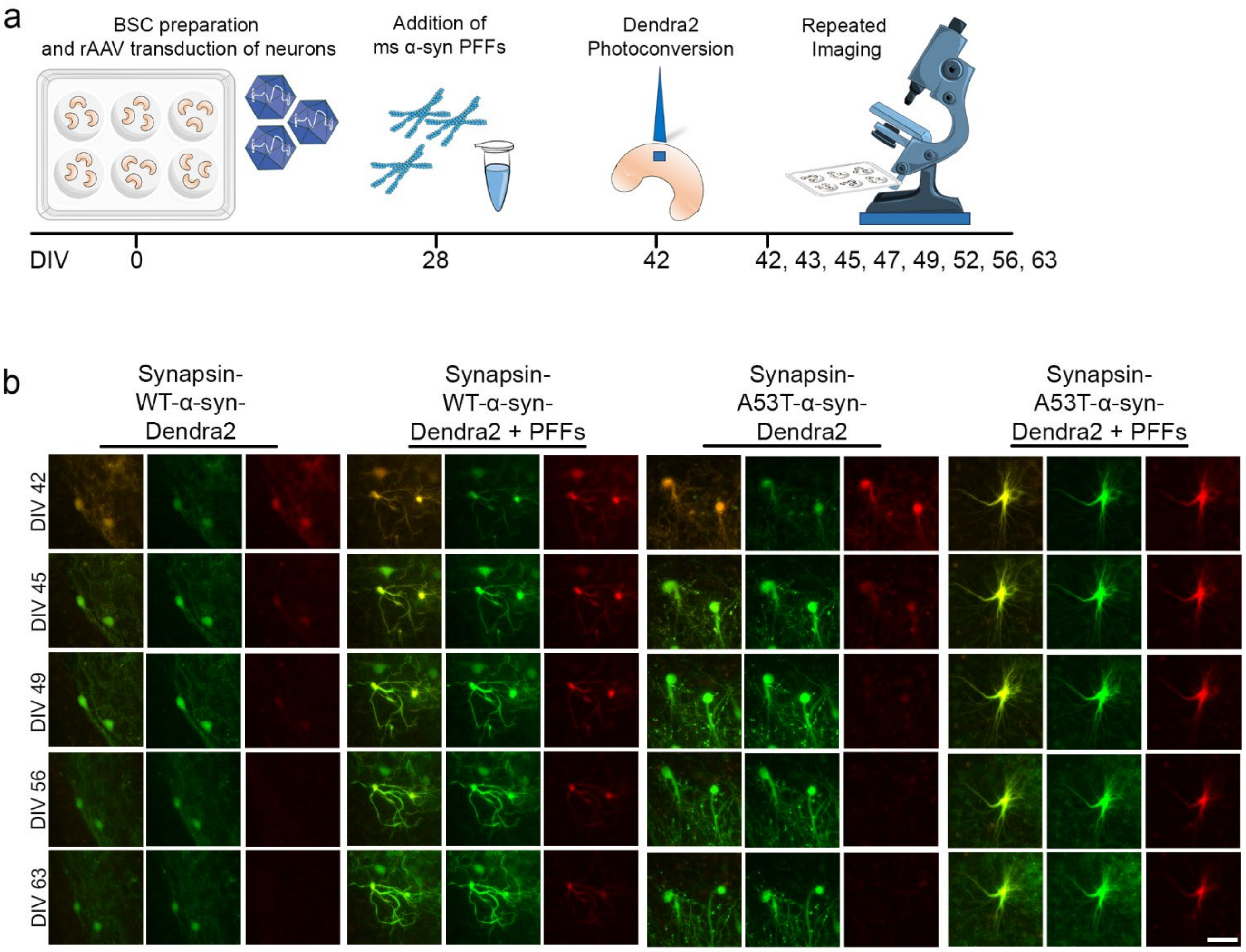

Supp. Figure 3: The addition of mouse α-syn PFFs slows WT and A53T-α-syn-Dendra2 turnover when α-syn is expressed in neurons

BSCs were transduced at 0 DIV with rAAVs to express WT-α-syn-Dendra2 or A53T-α-syn-Dendra2 under the Synapsin promoter, at 28 DIV mouse α-syn PFFs were added and then optical pulse labeling experiments began at 42 DIV. (A) Schematic diagram shows the timeline of the long-term optical pulse labeling experiments using photoconversion of Synapsin-Dendra2, Synapsin-WT-α-syn-Dendra2 and Synapsin-A53T-α-syn-Dendra2 at 42 DIV after seeding with PFFs at 28 DIV. (B) Representative images of photoconverted (red) and newly synthesized (green) Dendra2 above residual fluorescence in BSCs photoconverted at 42 DIV and imaged at several time points until 63 DIV reveals a proportion of neurons show long-lived photoconverted α-syn. Merge of both channels is also shown (n=6). Scale bar = 50 μm.

Supporting Information:
Figure 1A

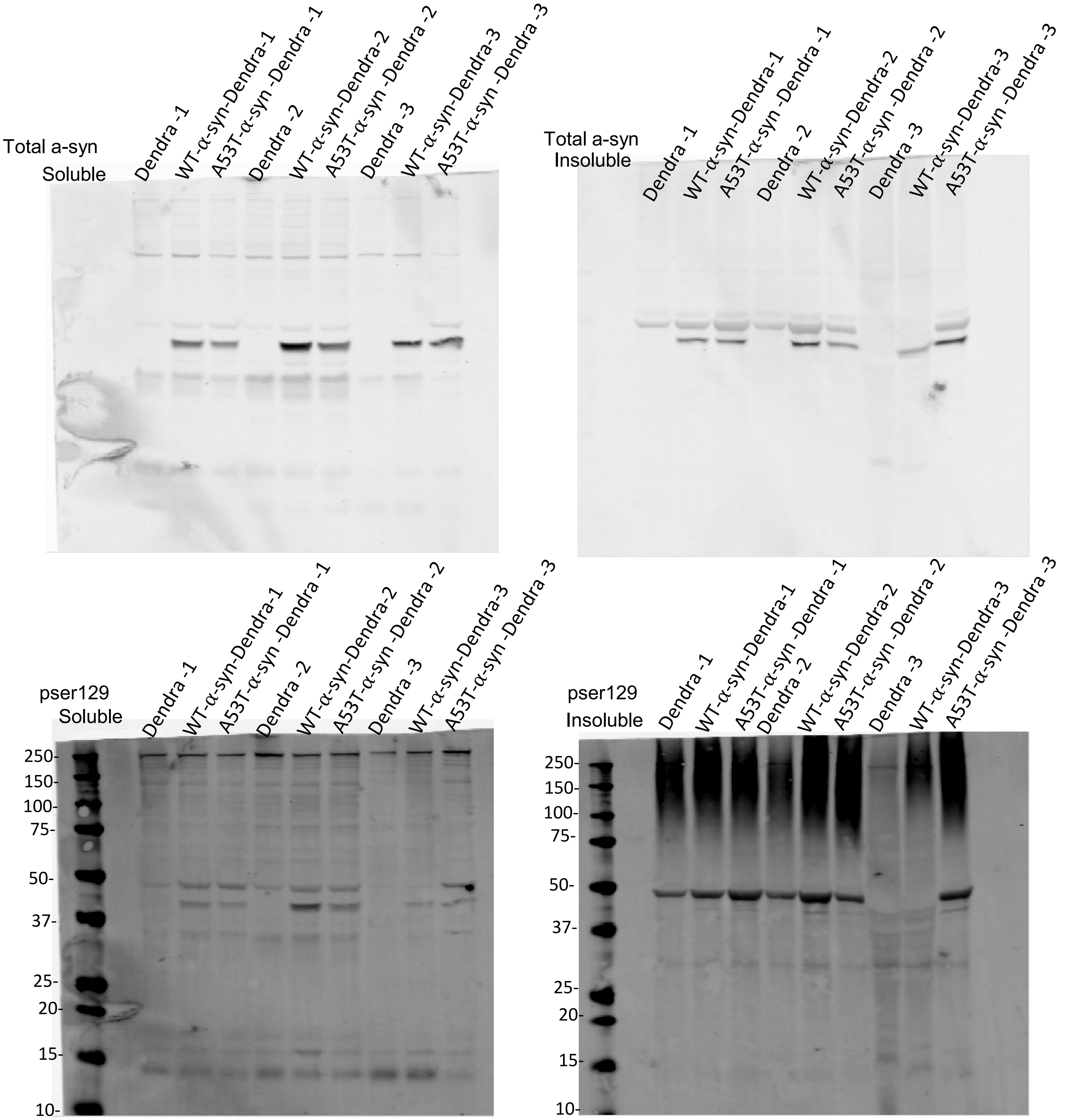

Supporting Information:
Figure 1C

Total α-Syn Soluble

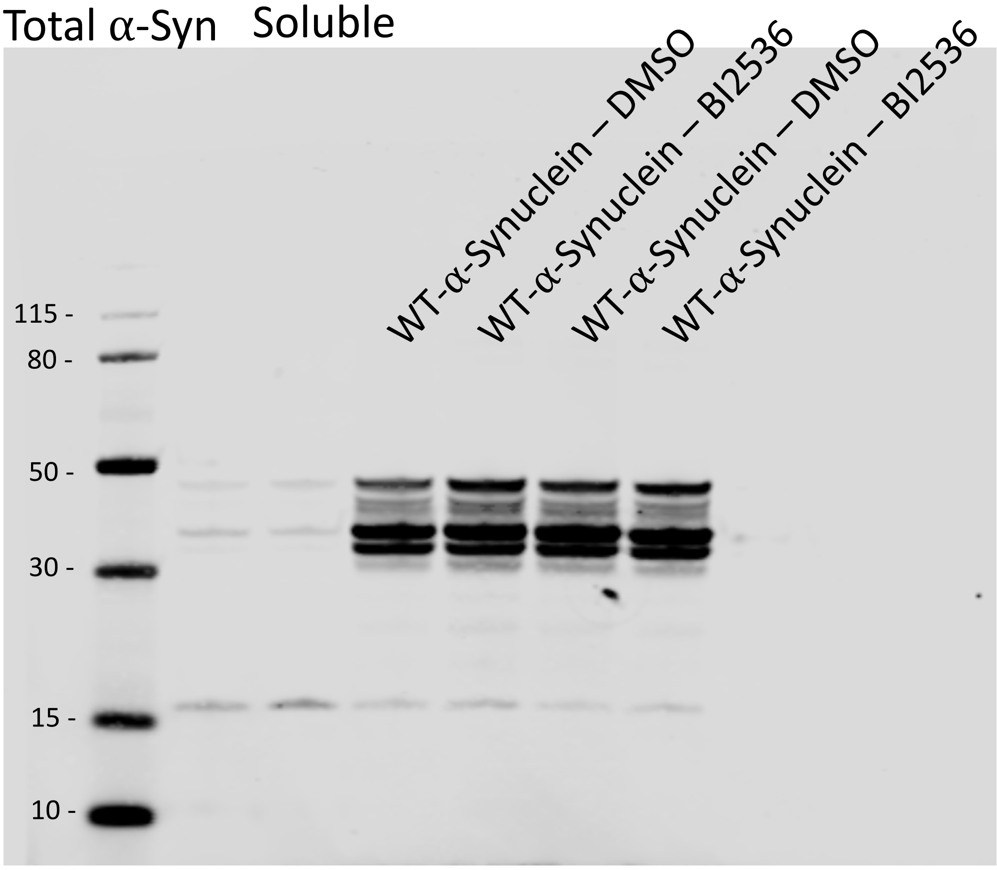

pser129 α-Syn Soluble

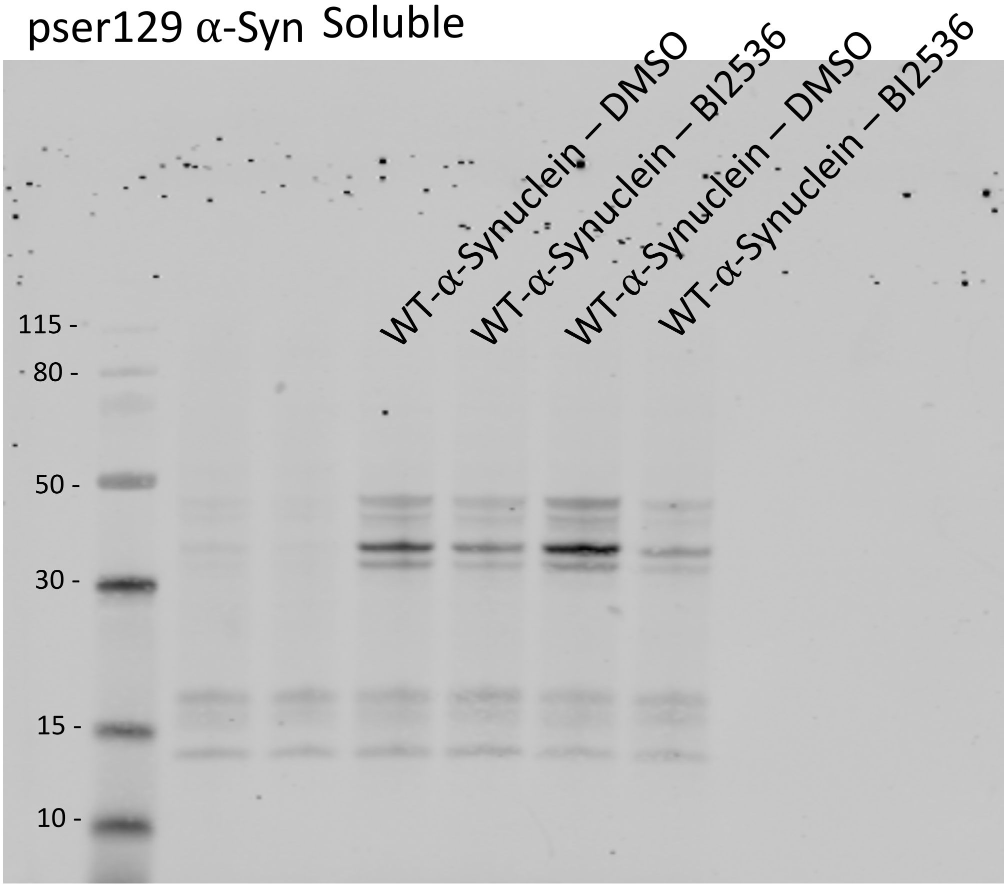

Supporting Information:
Figure 1C

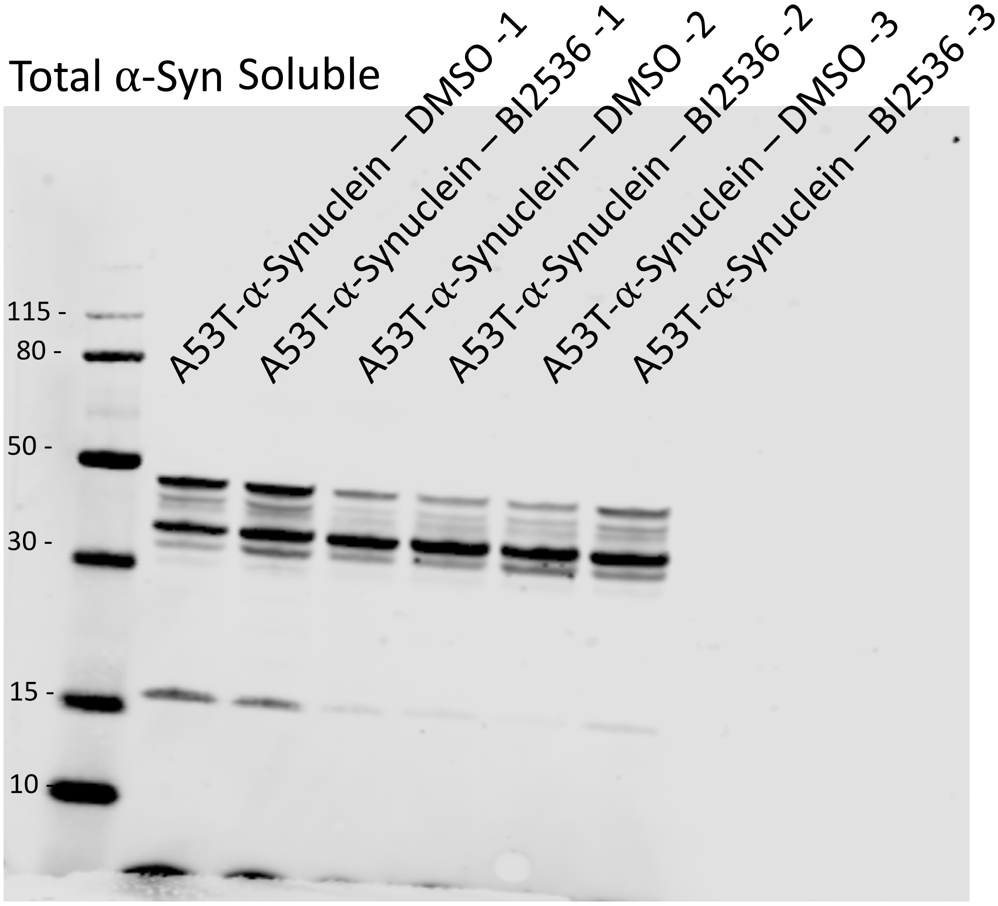

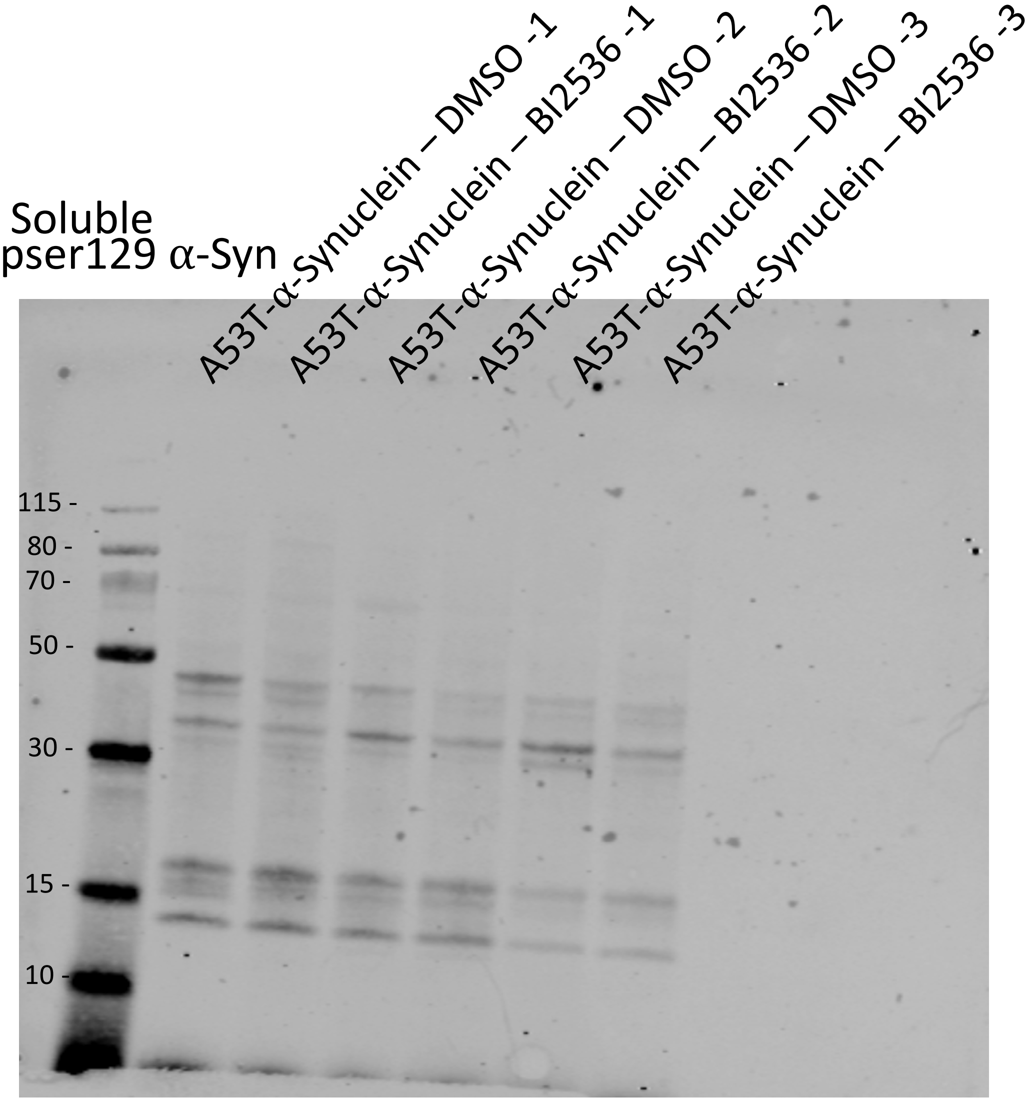

Supporting Information:
Figure 2A

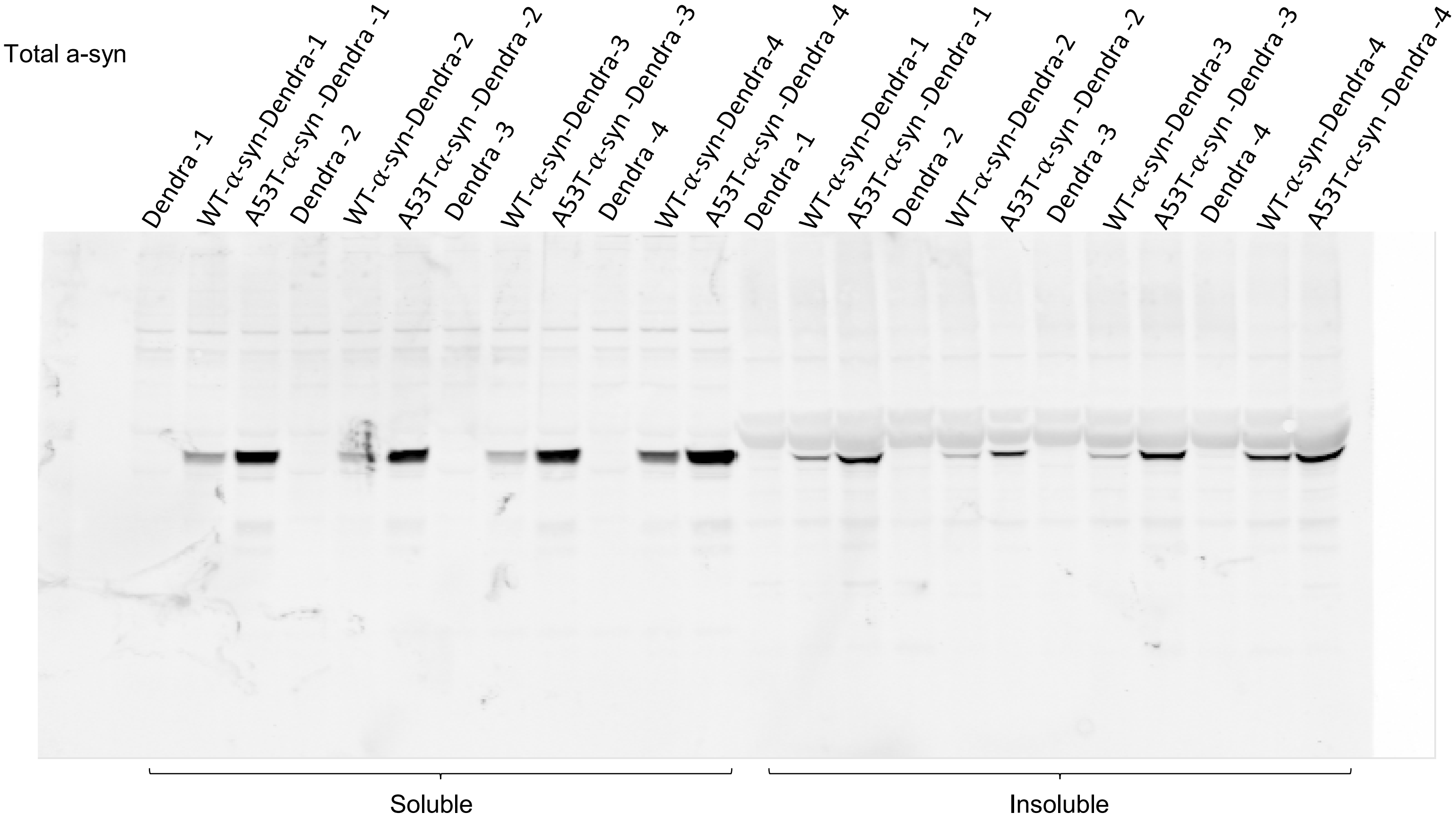

Supporting Information:
Figure 2A

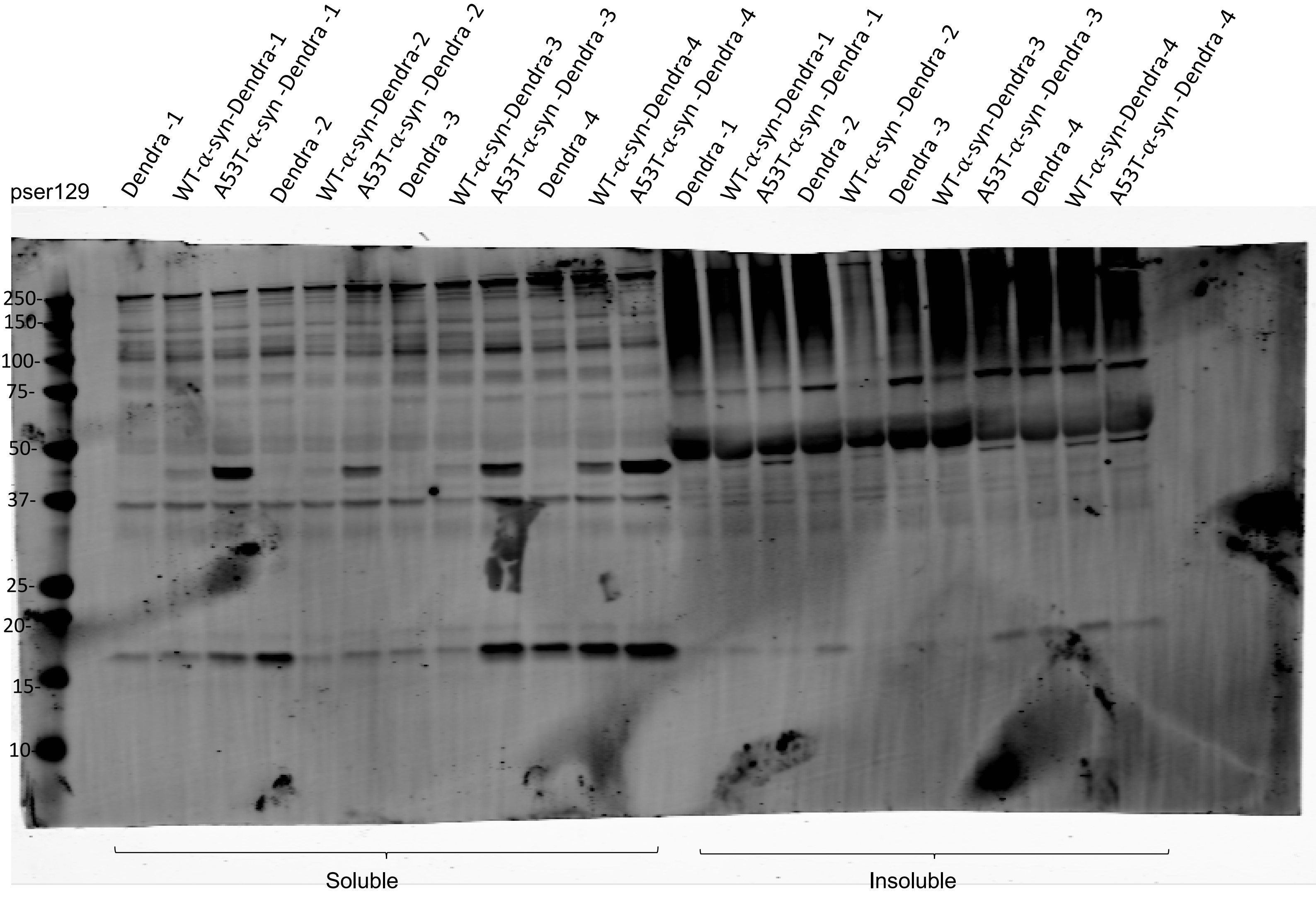

Supporting Information:
Figure 2B

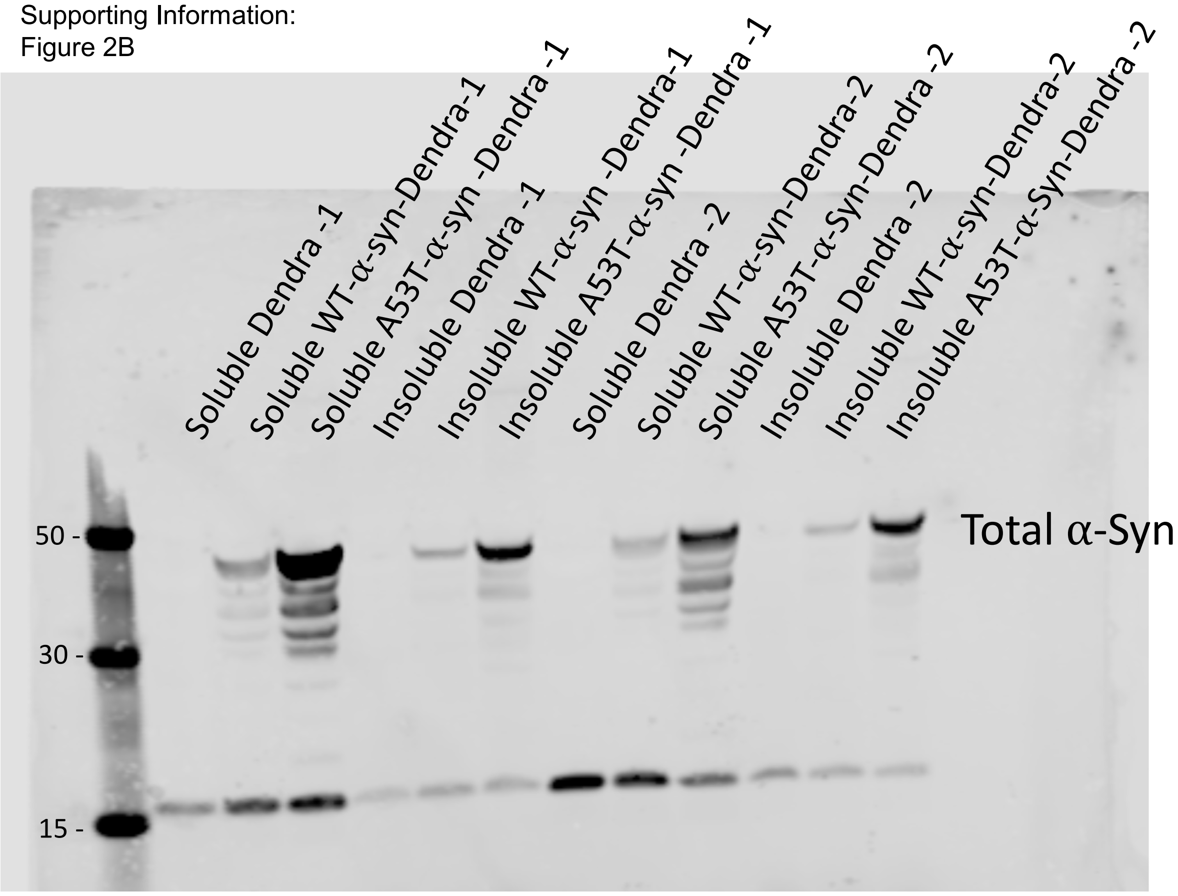

Supporting Information:
Figure 2C

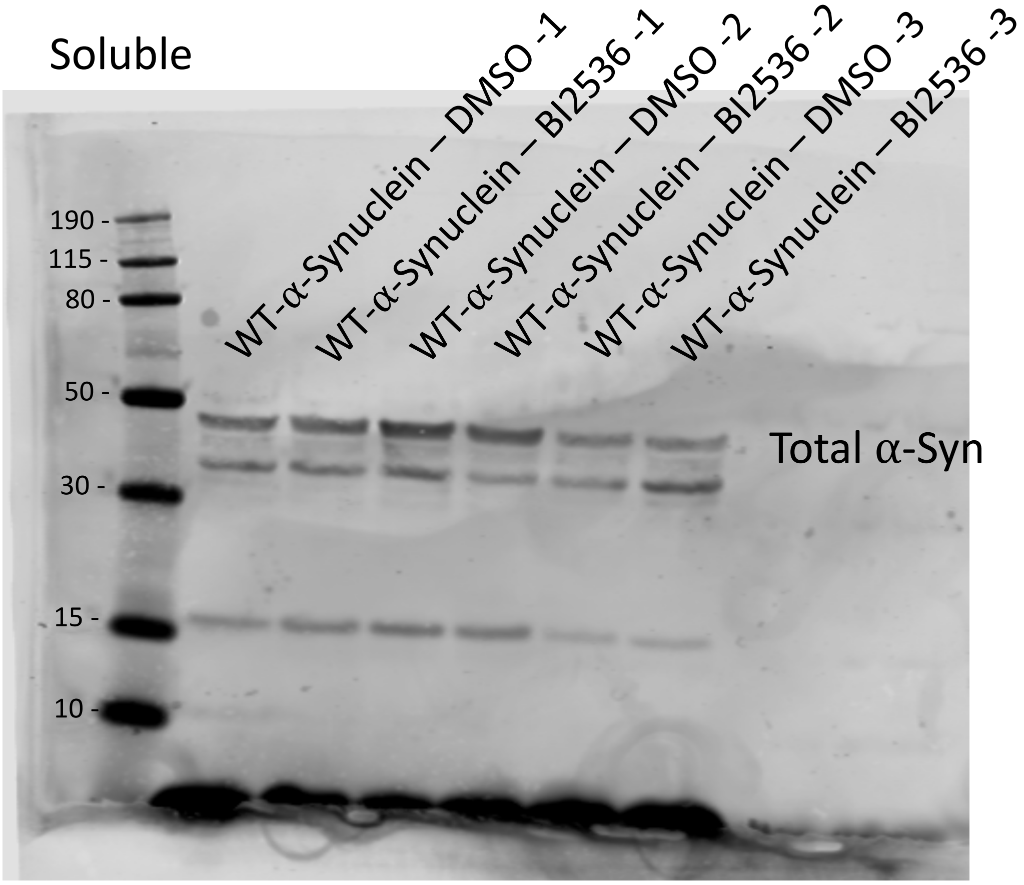

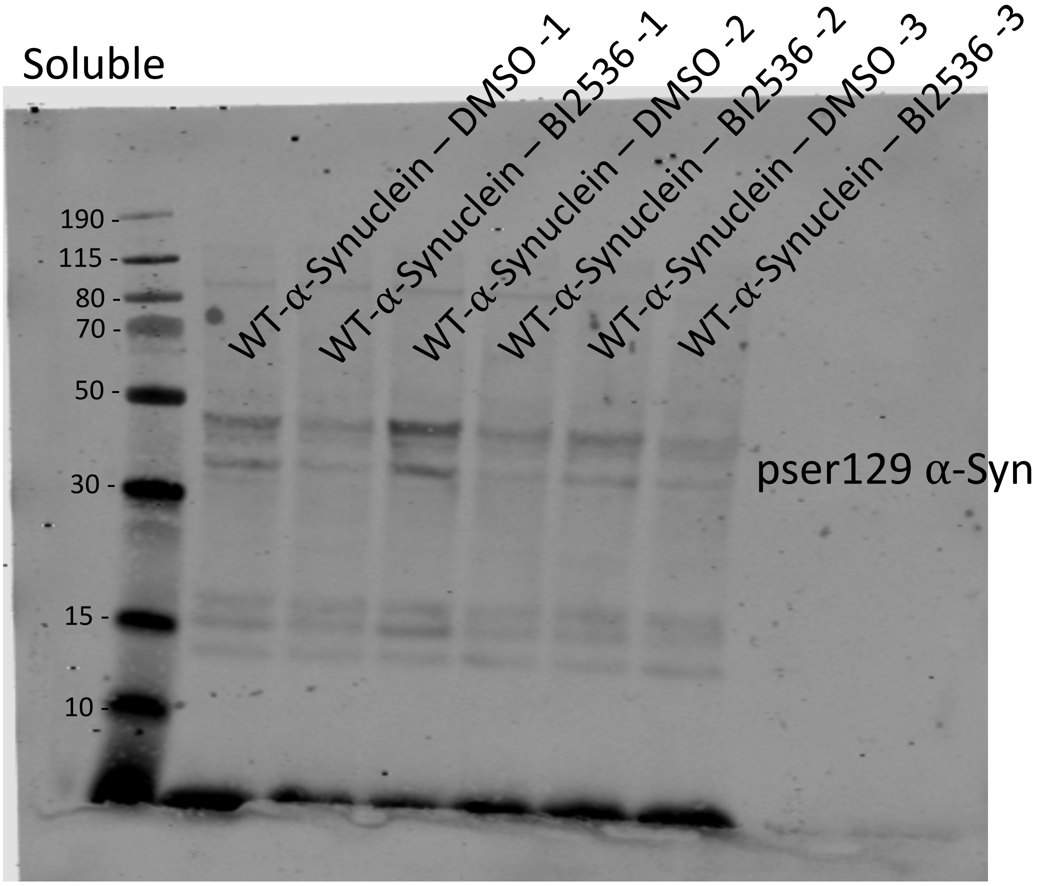

Supporting Information:
Figure 2C

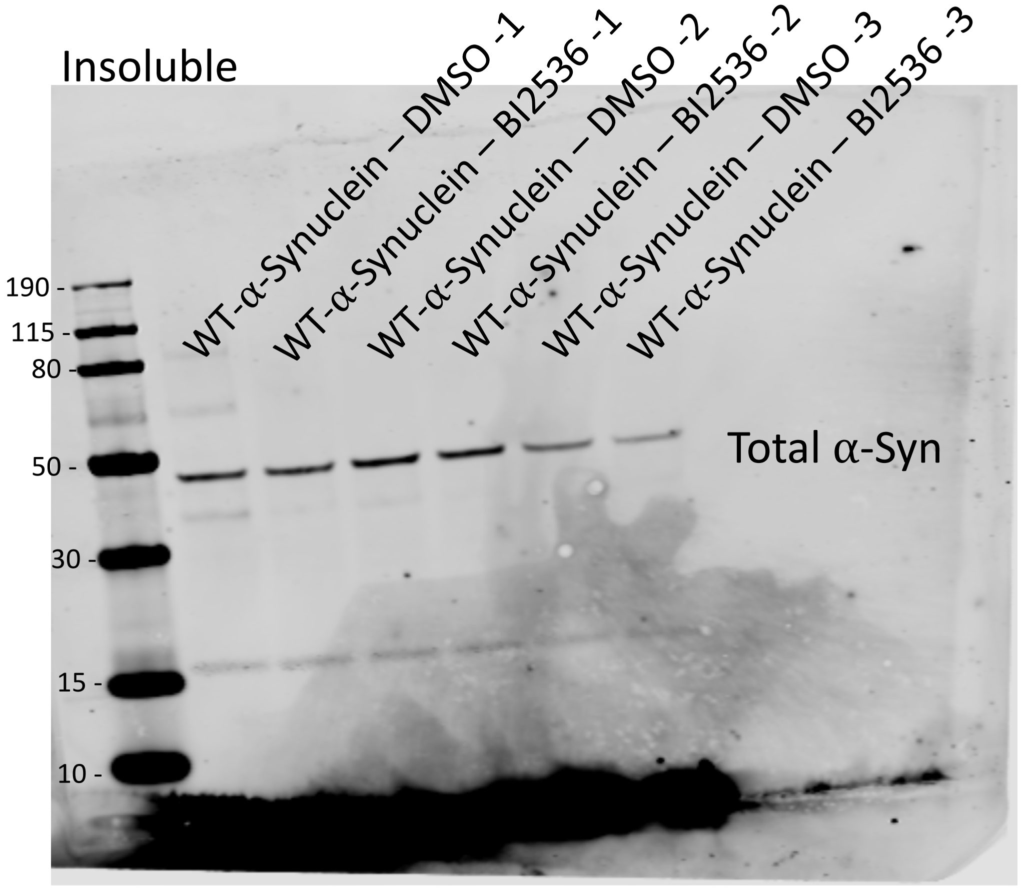

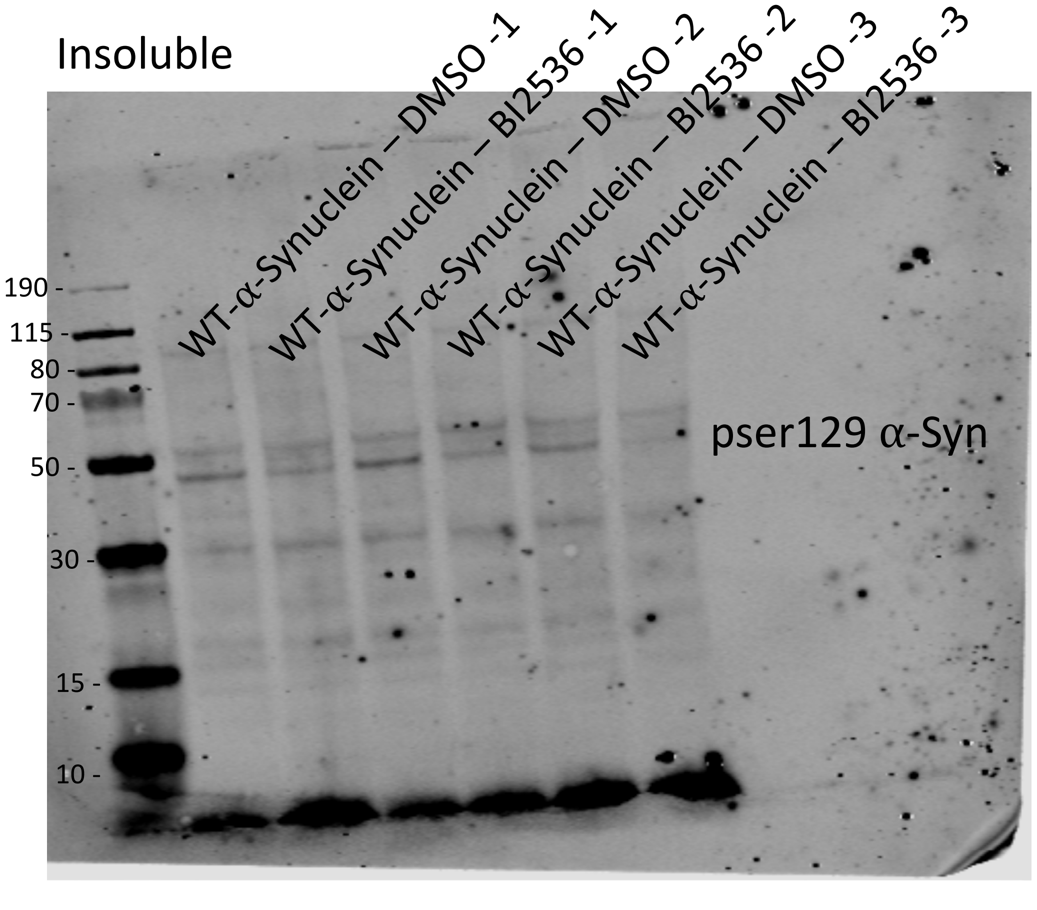

Supporting Information:
Figure 2C

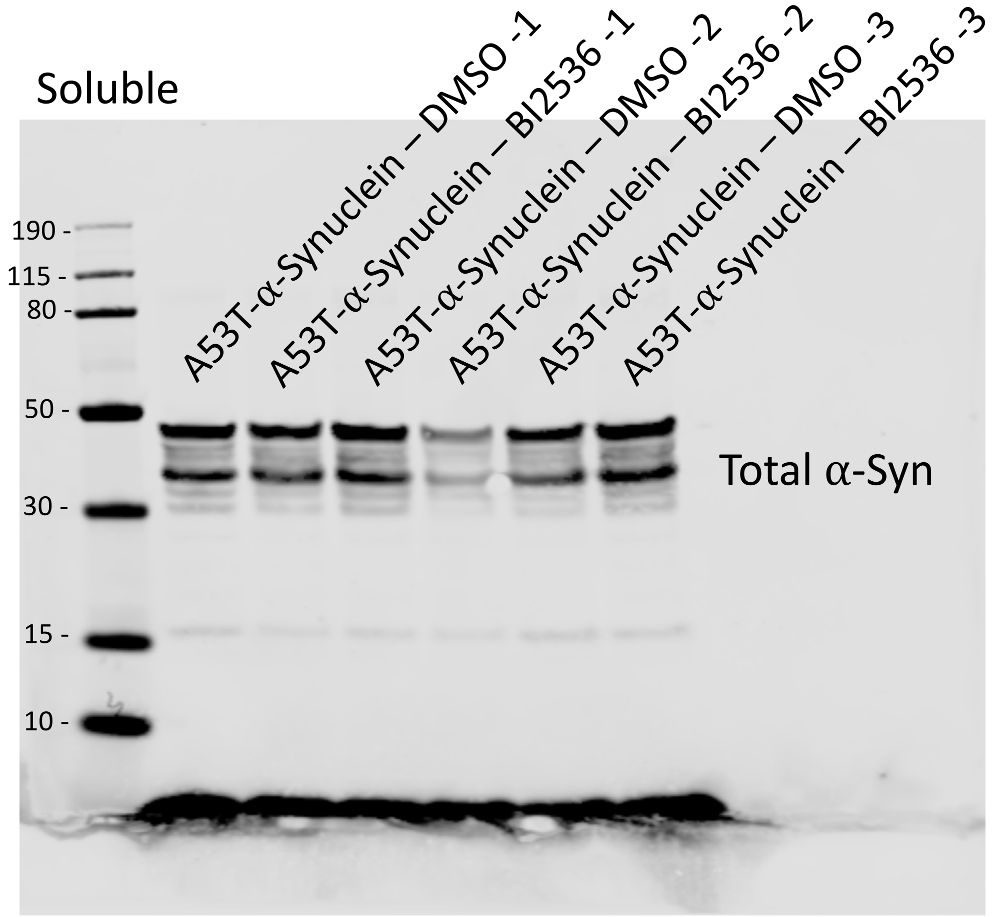

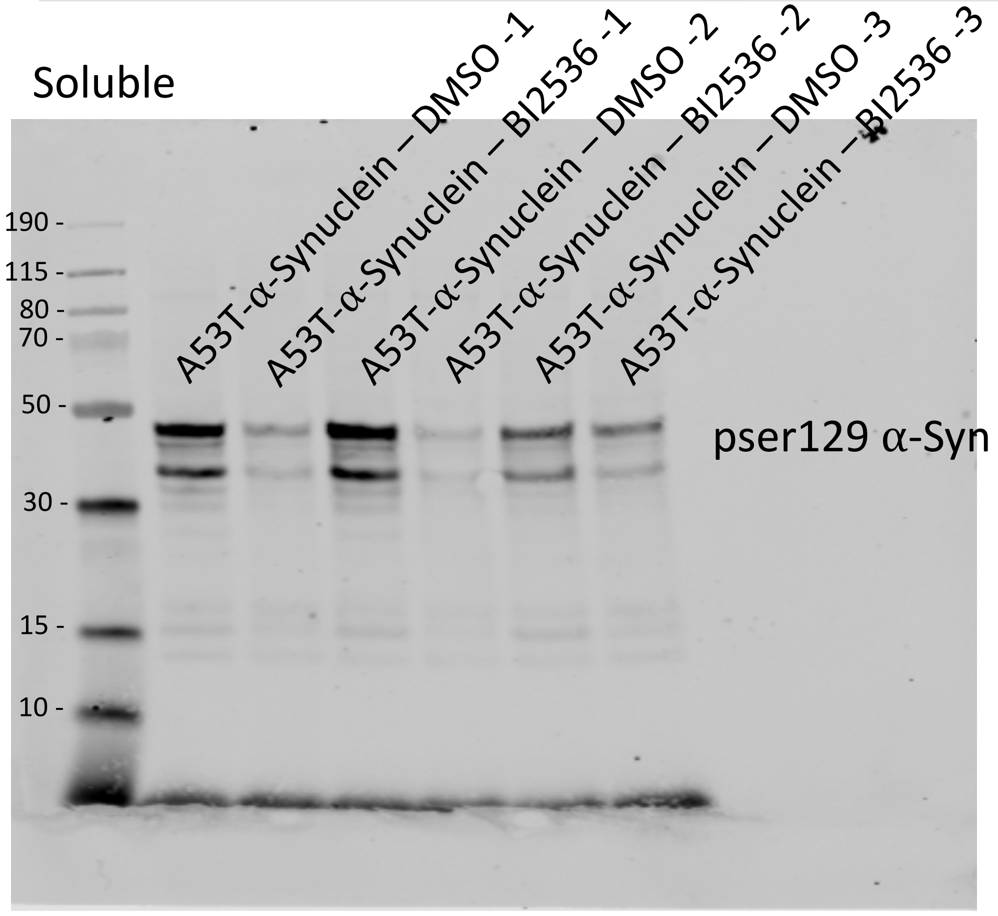

Supporting Information:
Figure 2C

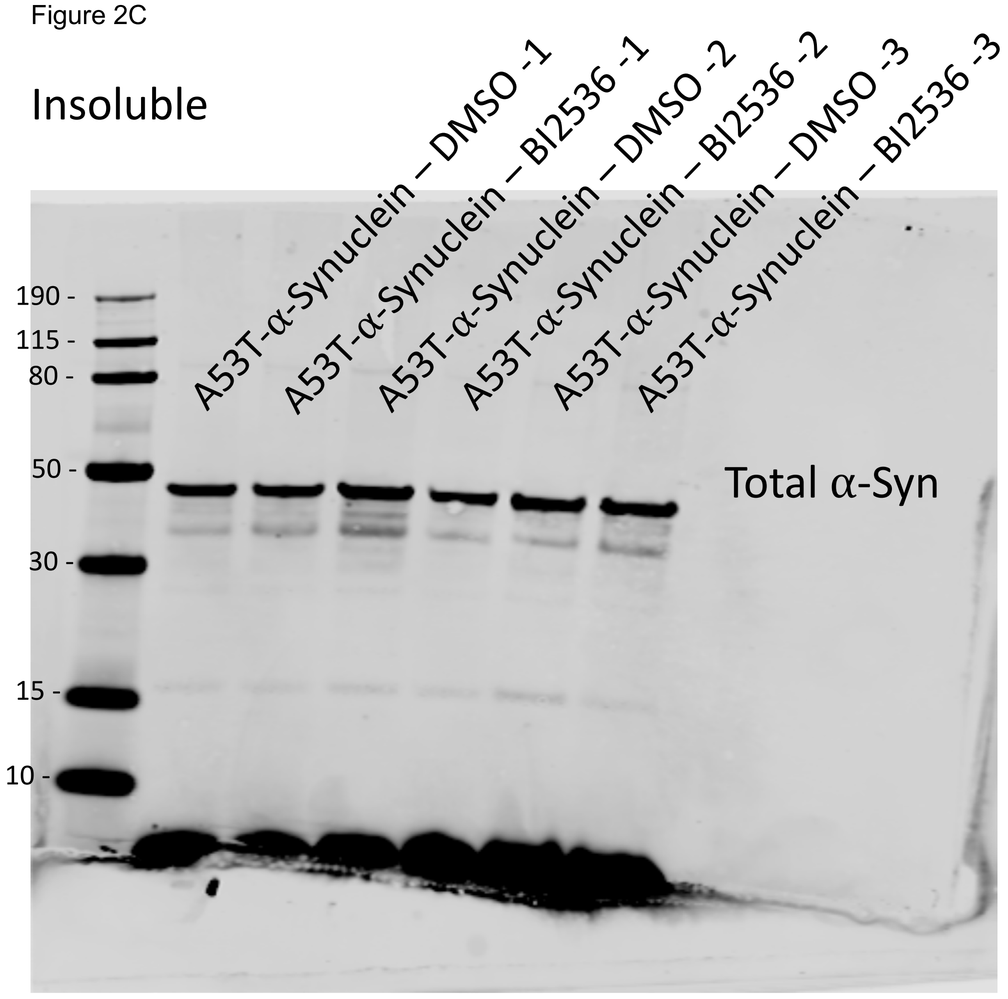

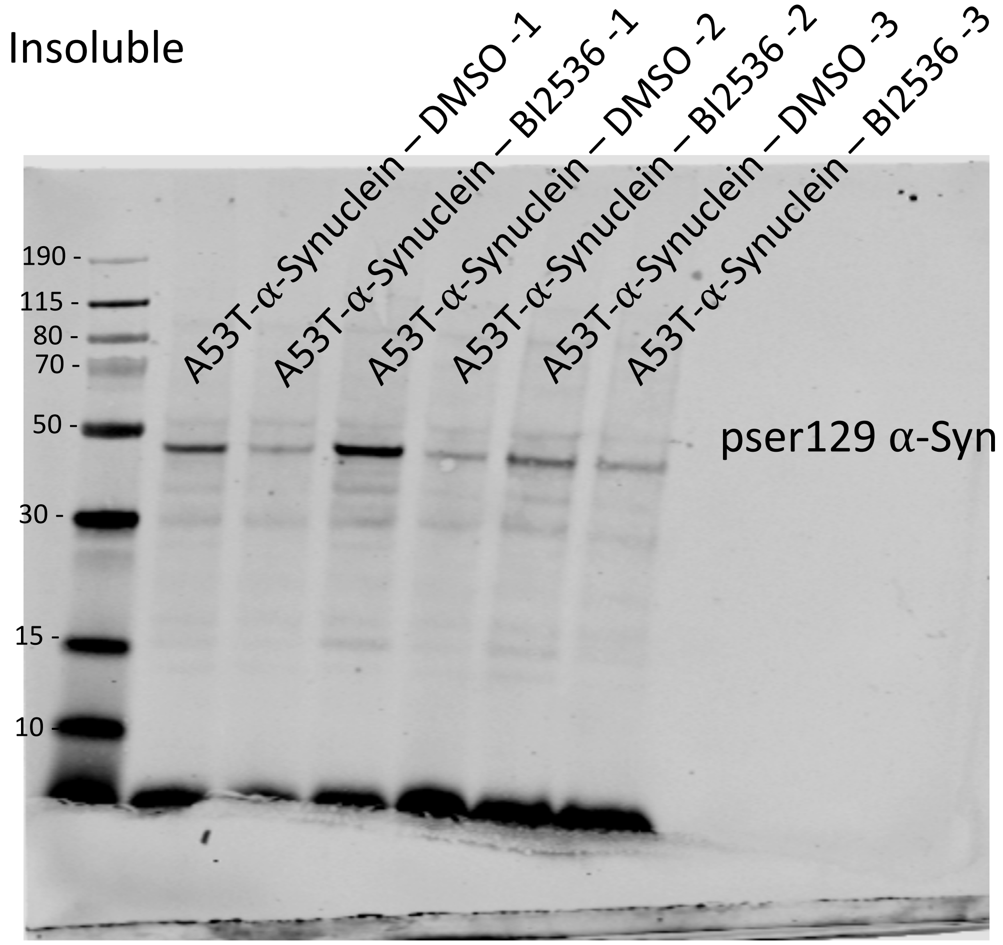

Supporting Information:
Figure 3A

Total a-syn
soluble

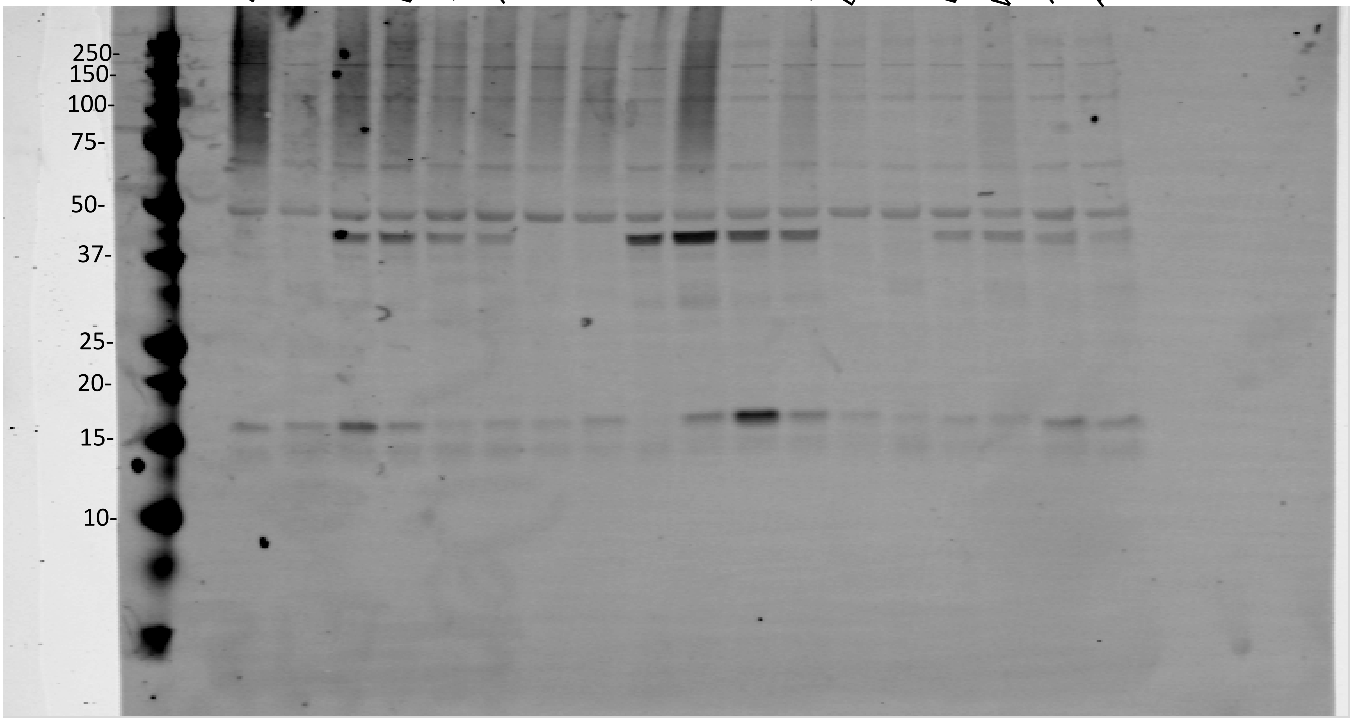

pser129
soluble

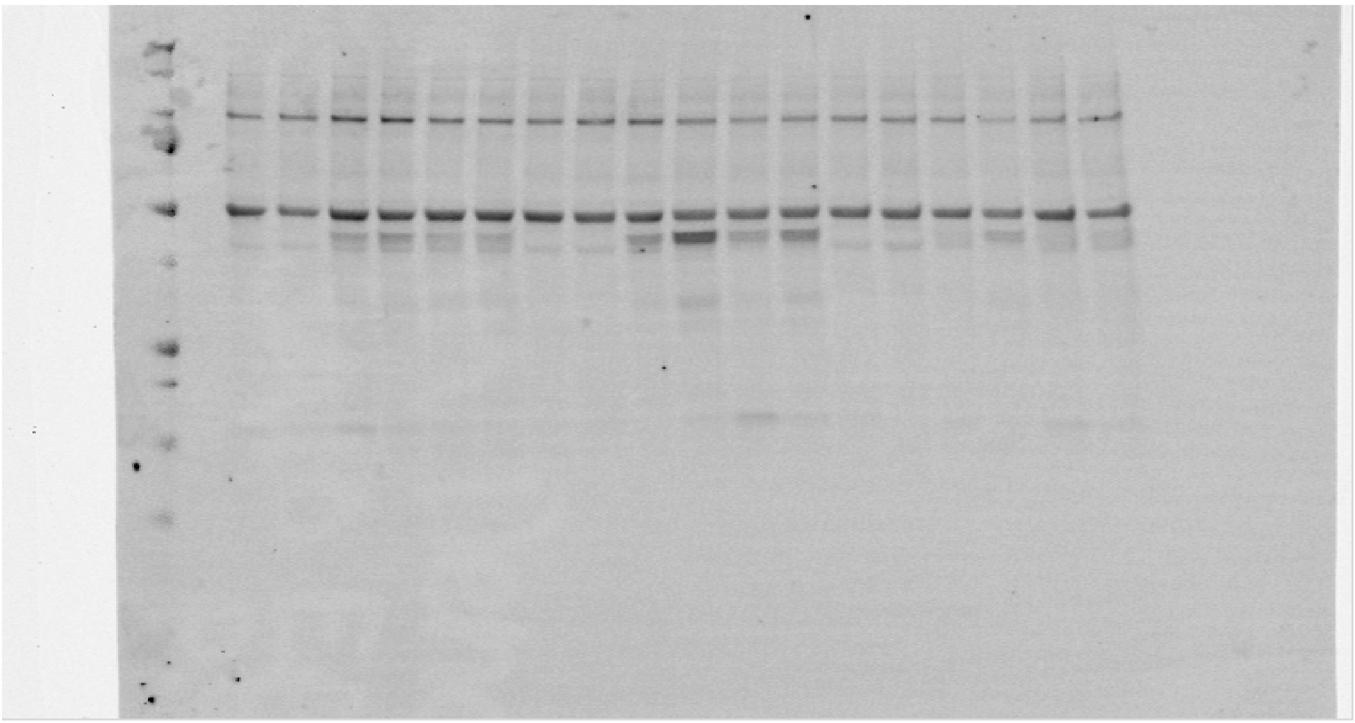

Supporting Information:
Figure 3A

Total a-syn
insoluble

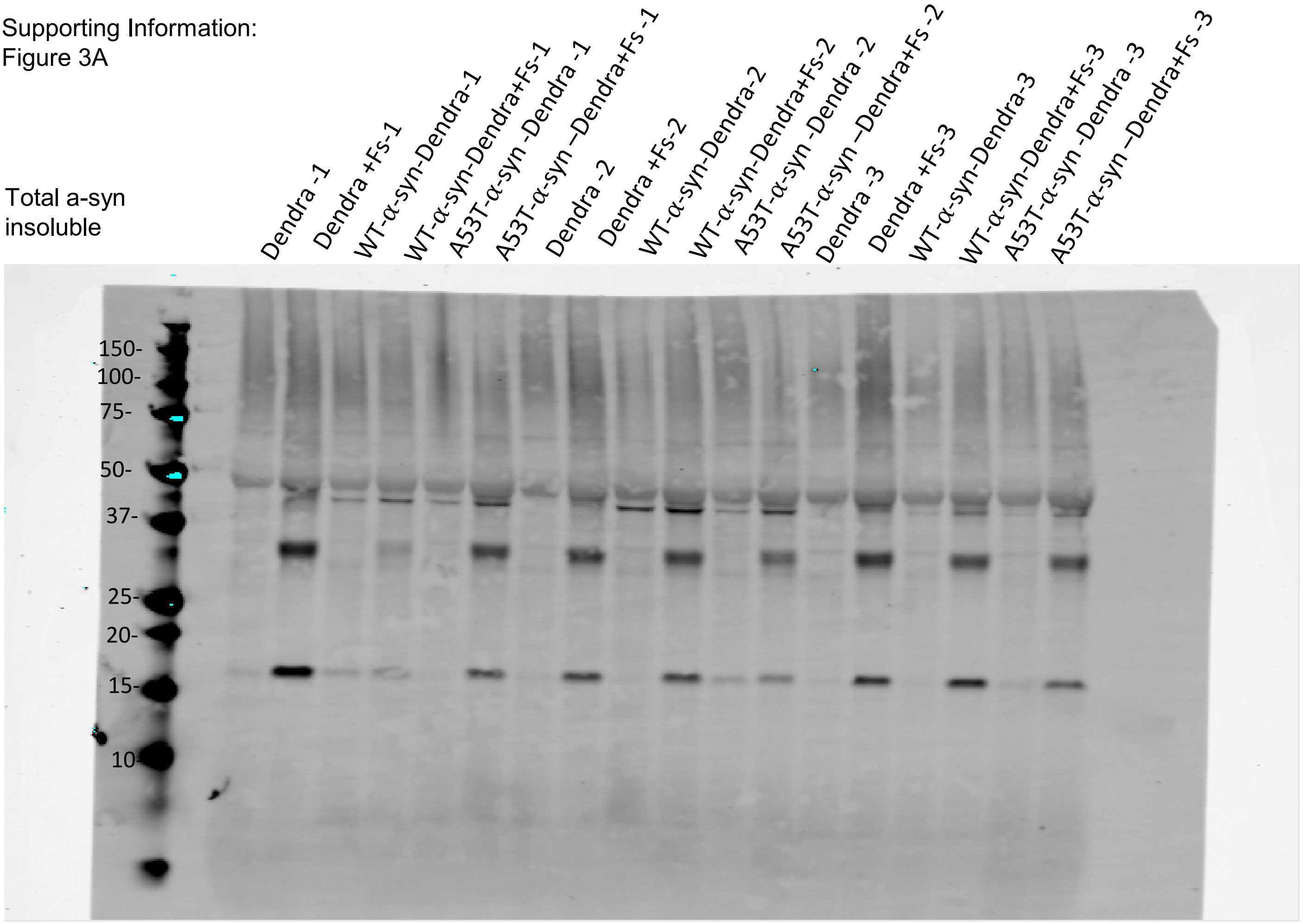

pser129
insoluble

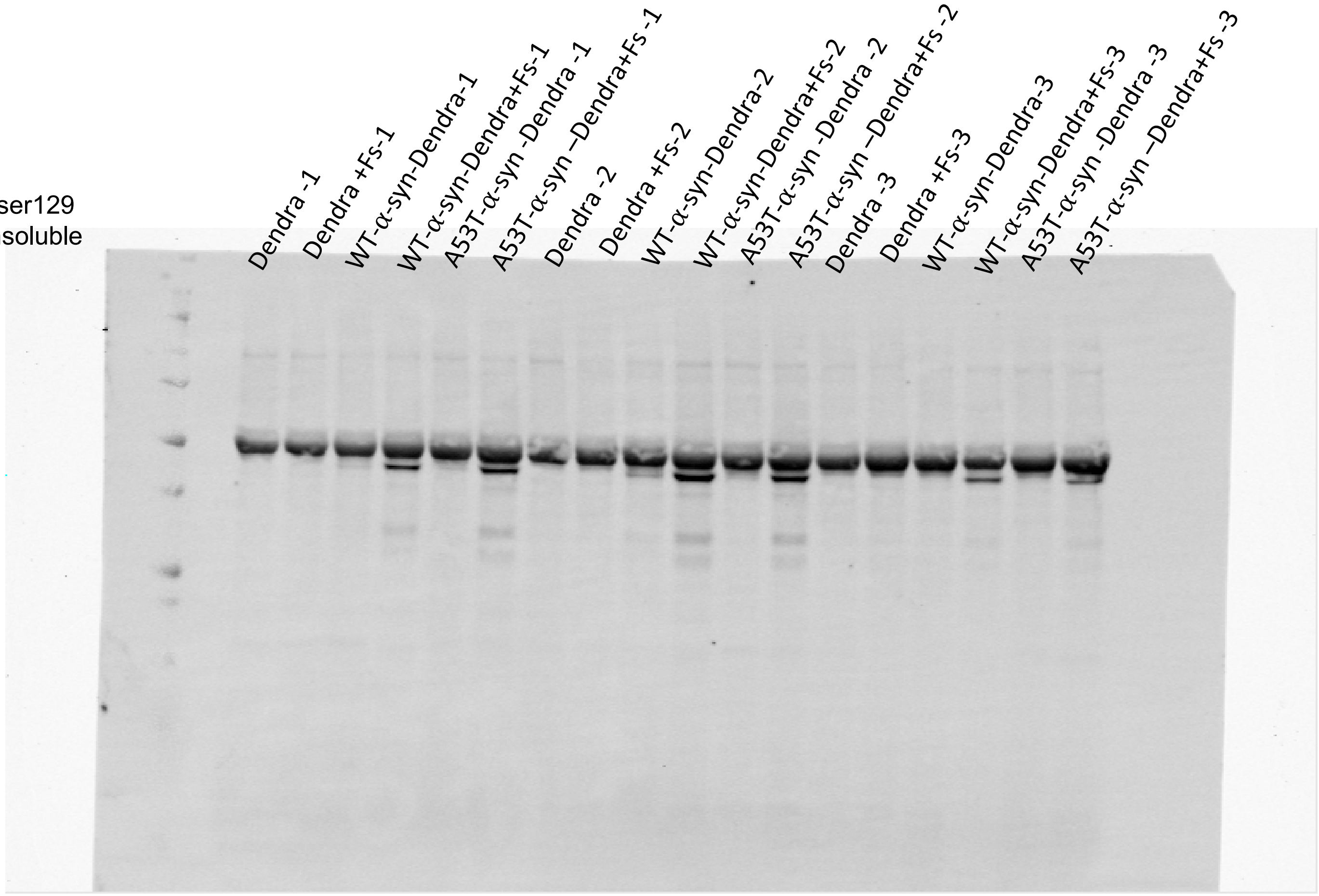

Supporting Information:
Supp Figure 1A

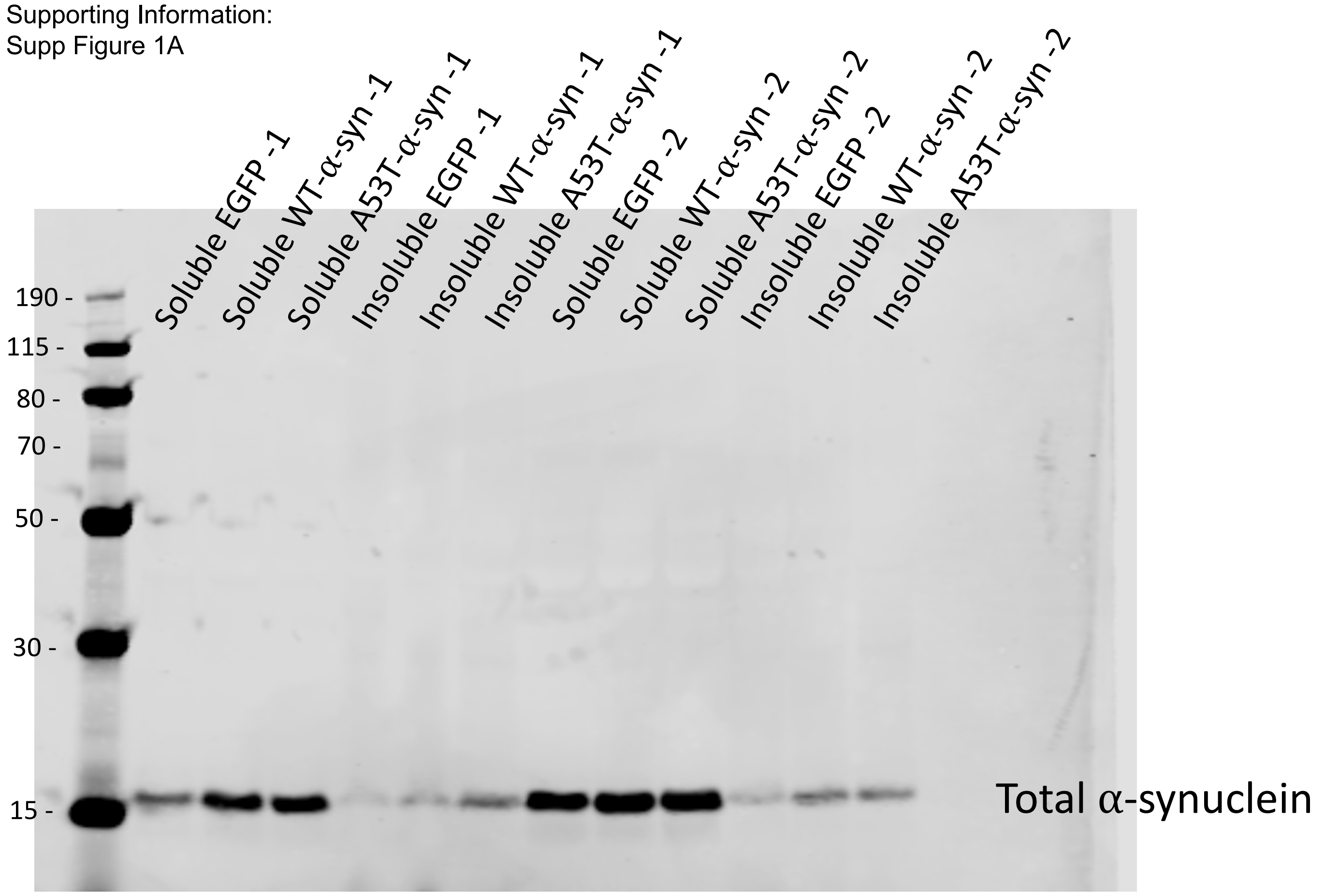

Supplement: Supplementary file 1 — Supplementary Information [file 41531_2022_434_MOESM1_ESM.pdf]
